# Two highly selected mutations in the tandemly duplicated *CYP6P4a* and *CYP6P4b* genes drive pyrethroid resistance in *Anopheles funestus* in West Africa

**DOI:** 10.1186/s12915-024-02081-y

**Published:** 2024-12-18

**Authors:** Nelly M. T. Tatchou-Nebangwa, Leon M. J. Mugenzi, Abdullahi Muhammad, Derrick N. Nebangwa, Mersimine F. M. Kouamo, Carlos S. Djoko Tagne, Theofelix A. Tekoh, Magellan Tchouakui, Stephen M. Ghogomu, Sulaiman S. Ibrahim, Charles S. Wondji

**Affiliations:** 1 Centre for Research in Infectious Diseases (CRID), P.O. BOX 13591, Yaounde, Cameroon; 2 Department of Biochemistry and Molecular Biology, Faculty of Science, University of Buea, P.O. Box 63, Buea, Cameroon; 3 Vector Biology Department, Liverpool School of Tropical Medicine (LSTM), Pembroke Place, Liverpool, L3 5QA UK; 4 Randall Center for Cell and Molecular Biophysics, Faculty of Life Sciences and Medicine, King’s College London, London, UK; 5 Syngenta Crop Protection, Werk Stein, Schaffhauserstrasse, Stein, CH4332 Switzerland; 6 Department of Biochemistry, Faculty of Science, University of Bamenda, Bamenda, Cameroon; 7 Centre for Biotechnology Research, Bayero University, Kano PMB, Kano 3011 Nigeria; 8 Department of Biochemistry, Bayero University, Kano PMB, Kano 3011 Nigeria

**Keywords:** *Anopheles funestus*, Insecticide resistance, Cytochrome P450, Insecticide metabolism assay, Heterologous expression, GAL4/UAS expression

## Abstract

**Background:**

Gaining a comprehensive understanding of the genetic mechanisms underlying insecticide resistance in malaria vectors is crucial for optimising the effectiveness of insecticide-based vector control methods and developing diagnostic tools for resistance management. Considering the heterogeneity of metabolic resistance in major malaria vectors, the implementation of tailored resistance management strategies is essential for successful vector control. Here, we provide evidence demonstrating that two highly selected mutations in *CYP6P4a* and *CYP6P4b* are driving pyrethroid insecticide resistance in the major malaria vector *Anopheles funestus*, in West Africa.

**Results:**

Continent-wide polymorphism survey revealed escalated signatures of directional selection of both genes between 2014 and 2021. In vitro insecticide metabolism assays with recombinant enzymes from both genes showed that mutant alleles under selection exhibit higher metabolic efficiency than their wild-type counterparts. Using the GAL4-UAS expression system, transgenic *Drosophila* flies overexpressing mutant alleles exhibited increased resistance to pyrethroids. These findings were consistent with in silico predictions which highlighted changes in enzyme active site architecture that enhance the affinity of mutant alleles for type I and II pyrethroids. Furthermore, we designed two DNA-based assays for the detection of CYP6P4a-M220I and CYP6P4b-D284E mutations, showing their current confinement to West Africa*.* Genotype/phenotype correlation analyses revealed that these markers are strongly associated with resistance to types I and II pyrethroids and combine to drastically reduce killing effects of pyrethroid bed nets.

**Conclusions:**

Overall, this study demonstrated that *CYP6P4a* and *CYP6P4b* contribute to pyrethroid resistance in *An. funestus* and provided two additional insecticide resistance molecular diagnostic tools that would contribute to monitoring and better management of resistance.

**Supplementary Information:**

The online version contains supplementary material available at 10.1186/s12915-024-02081-y.

## Background

Globally, malaria continues to be a leading cause of human mortality, disproportionately affecting sub-Saharan Africa. In 2022, an estimated 233 million cases were reported, resulting in 608 thousand deaths, with more than 90% occurring in sub-Saharan Africa and in children under the age of 5 [1]. The control of the disease heavily relies on the use of insecticide-based vector control tools, primarily long-lasting insecticide-treated bed nets (LLINs) and indoor residual sprays (IRS) [2] which have achieved an unprecedented reduction in the disease burden, accounting for about 78% of the decline in malaria cases between 2000 and 2015 [3, 4]. Unfortunately, the massive use of insecticides has exerted a selective pressure on mosquito populations, leading to the emergence of resistance mechanisms that enable mosquitoes to survive exposure to these chemicals.

Resistance to all primary insecticide classes currently used in public health (pyrethroids, organochlorines, carbamates, and organophosphates) has been widely reported in the dominant African malaria vectors [5]. Specifically, resistance to pyrethroids in *An. funestus* has been extensively reported in Sub-Saharan Africa, including Southern Africa [6–8], East Africa [9–11], Central Africa [12, 13], and West Africa [14–16]. The resistance to pyrethroids poses a significant challenge in malaria control efforts, given that this is the primary insecticide class recommended by WHO for treating bed nets [1]. The efficacy of pyrethroid-treated bed nets is notably compromised by insecticide resistance [17–19], consequently contributing to the persistent transmission of malaria. An illustrative instance is the upsurge of malaria in the Kwazulu/Natal province in South Africa between 1996 and 2001, which was attributed to the emergence of pyrethroid resistance in *An. funestus* Giles. [20]. Consequently, it is crucial to understand the genetic mechanisms behind insecticide resistance and develop tools for early detection and mitigation of resistance. These efforts are essential for formulating effective strategies to manage resistance in malaria vectors [21, 22].

The main mechanism of insecticide resistance in *An. funestus* is metabolic resistance, characterised by mosquitoes’ increased overexpression of key detoxification genes (glutathione S-transferases (GSTs), cytochrome p450s, and esterases (ESTs)) that metabolise the insecticides before they reach their targets [23, 24]. While significant progress has been made in understanding target-site resistance, with key mutations identified over 25 years ago in *An. gambiae* [25], unravelling the complexities of metabolic resistance has proven to be more complex. This complexity arises from the involvement of multiple gene families, encompassing dozens of genes, with an estimated total of around 200 genes potentially contributing to the phenotype [23, 24]. Furthermore, even after identifying candidate resistance genes, numerous resistance pathways exist, including mutations in regulatory and coding regions and transposable elements [26]. Consequently, detecting genetic variants responsible for metabolic resistance and establishing diagnostic tools though pivotal are challenging. Nonetheless, significant progress has been made with the detection of the first DNA-based maker in the GST gene *GSTe2* conferring DDT resistance [27] in *An. funestus*. Similarly, the elucidation of the *cis*-regulatory elements associated with pyrethroid resistance in southern Africa led to the development of the first DNA-based markers for cytochrome P450-mediated (*CYP6P9a* and *CYP6P9b*) metabolic resistance in *An. funestus* [18, 19]. However, it is worth noting that in this vector, metabolic resistance to pyrethroids displays heterogeneity across Africa, with different genes driving resistance in different regions due to barriers to gene flow. Notably, in Southern Africa, *CYP6P9a* and *CYP6P9b* have been identified as drivers of metabolic resistance, while in East Africa, resistance is driven by *CYP9K1*, and in Central Africa by *CYP325A*, via mechanisms of overexpression and allelic variations that enhance enzyme detoxification activity [17–19, 28, 29]. This emphasises the need to establish specific molecular diagnostic tools to contribute to the implementation of tailored resistance management strategies [30–32]. In this context, the tandemly duplicated cytochrome P450 genes, *CYP6P4a* and *CYP6P4b*, have been consistently reported to be the most over-expressed genes in pyrethroid-resistant populations of *An. funestus* in West Africa [15, 18, 19]. However, their direct involvement in resistance and the associated variants have not been identified, hindering the early detection and monitoring of resistance in these populations. In this study, we conducted an Africa-wide temporal polymorphism analysis of the duplicated *CYP6P4a* and *CYP6P4b* genes, revealing heightened signatures of directional selection of predominant mutant alleles between 2014 and 2021. Through in silico modelling and molecular docking, we predicted a greater affinity of the selected alleles to pyrethroids. Moreover, in vitro insecticide metabolism assays with recombinant enzymes and in vivo transgenic expression of *CYP6P4a* and *CYP6P4b* in *Drosophila melanogaster* flies followed by insecticide-contact bioassays demonstrated that the selected alleles of both genes exhibited higher ability to metabolise both type I and II pyrethroids, resulting in significantly increased survivorship compared to the wild-type alleles. In addition, we have developed two DNA-based resistance diagnostic tools that will allow the spatiotemporal tracking of the spread of these resistance-conferring alleles across the continent. We also show here that both CYP6P4a-M220I and CYP6P4b-D284E mutations are robust markers that predict resistance status co-dominantly, revealing a significant reduction in the killing effect of pyrethroid-only bed nets. Currently, these markers have been detected in West Africa and were found absent in other regions. Taken together, the findings of this study will guide the implementation of the more effective vector control interventions that aim to limit the selection and spread of the resistance alleles to other regions of Africa.

## Results

### Africa-wide polymorphism analysis of CYP6P4a and CYP6P4b reveals signatures of directional selection

**Fig. 1:**
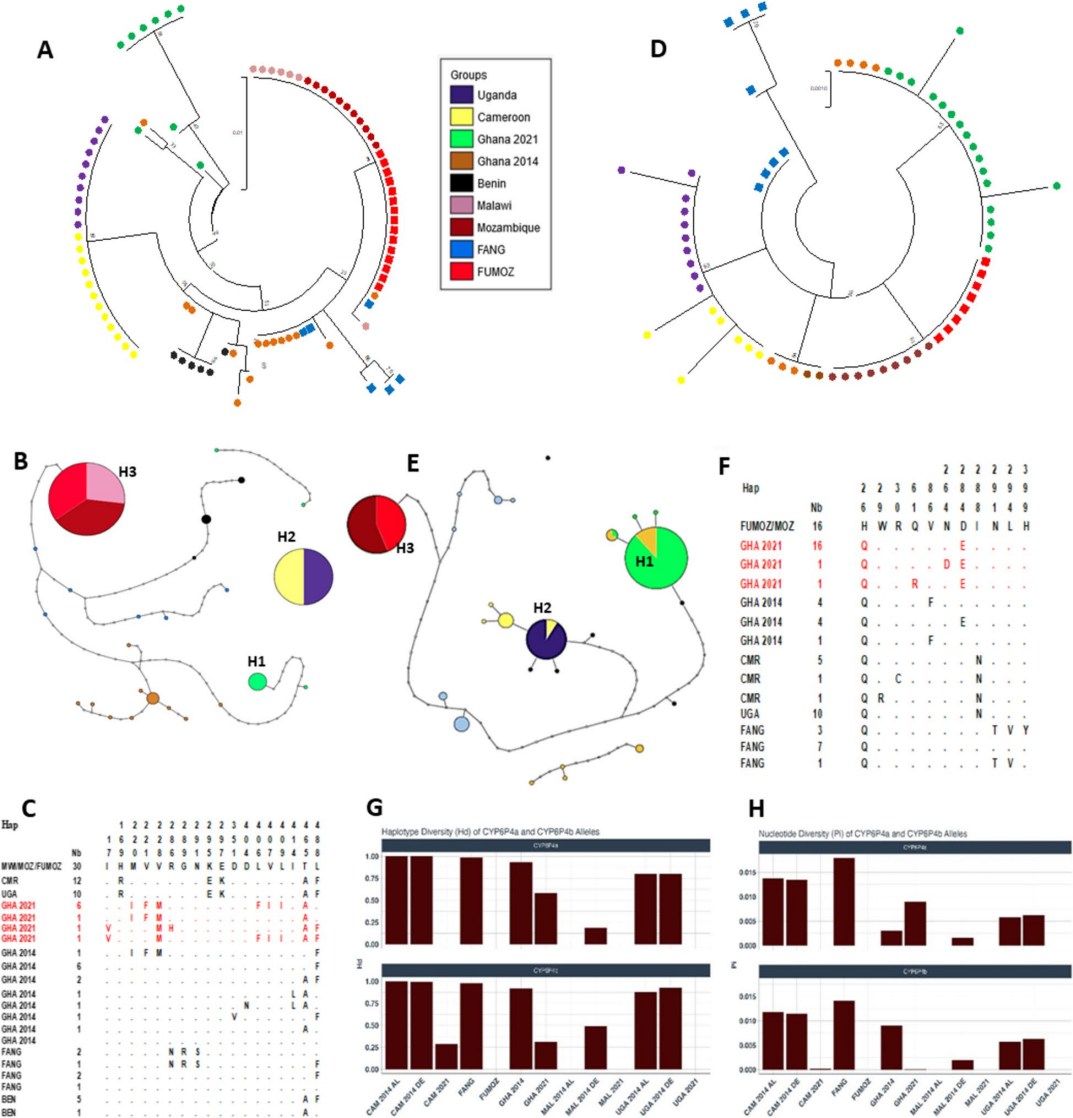
Schematic representation of haplotypes and genetic diversity of *CYP6P4a* and *CYP6P4b* in resistant mosquitoes across Africa, FUMOZ, and FANG. **A** Phylogenetic tree for *CYP6P4a* plotted using the JTT model, showing selection of major alleles in all regions in 2021. **B** Haplotype network for *CYP6P4a* revealing a fixed haplotype common Southern Africa, a fixed haplotype common to Uganda and Cameroon, and major haplotypes in Ghana and Benin, but high diversity in FANG. **C** Schematic representation of haplotypes of *CYP6P4a* with mutations occurring in Ghana highlighted in red. **D** Phylogenetic tree for *CYP6P4b* plotted using the JTT model, showing selection of major alleles in all regions in 2021. **E** *CYP6P4b* haplotype network. A fixed haplotype is observed in Southern population (Malawi and FUMOZ) and the formation of a major haplotype common to Uganda and Cameroon. **F** Schematic representation of haplotypes of *CYP6P4b* with D284E mutation occurring in Ghana highlighted in red. **G** Plot of genetic diversity parameters of *CYP6P4a and CYP6P4b* across Africa showing the signatures of a directional selection of *CYP6P4a and CYP6P4b* between 2014 and 2021 in haplotype diversity (Hd) and nucleotide diversity (*π*)

Africa-wide polymorphism analysis showed a high level of homogeneity and reduced diversity of *CYP64a* and *CYP6P4b* within individual populations but with significant variation between geographical regions (Additional File 2: Table S1). The *CYP6P4a* gene had a total of 24 haplotypes with 84 polymorphic sites (Additional File 2: Table S1). The resistant Ghanaian mosquito sequences showed reduced haplotype diversity (Hd = 0.583) but with a high number of polymorphic sites which were from a singleton that differed significantly from the predominant haplotype under selection. Amino acid phylogenetic tree analysis revealed that the majority of Ghana sequences clustered together and were distinct from other populations (Fig. 1A). The haplotype network also indicated the presence of a major haplotype (H1) (Fig. 1B) circulating in the population, with 6 out of 9 sequences harbouring it (Fig. 1C). Benin sequences also showed the existence of a major haplotype (5/6) that clustered away from the Ghanian population although both are in West Africa (Fig. 1B). Similarly, *CYP6P4b* demonstrated a reduction in diversity across all resistant populations. A total of 24 haplotypes were identified, encompassing 69 polymorphic sites of which 16 were non-synonymous mutations (Additional File 2: Table S1). Again, the Ghanaian population displayed a significant reduction in diversity, characterised by two non-synonymous mutations, a low haplotype diversity (Hd) of 0.216, and a low nucleotide diversity (π 0.00014) (Additional File 2: Table S1). Phylogenetic analyses confirmed the distinct clustering of all Ghanaian sequences from other populations (Fig. 1D) and the prevalence of a predominant haplotype (H1) present among 16 out of 18 sequences (Fig. 1E). For both genes, the southern populations displayed no diversity (Hd = 0, D = 0, π = 0) (Additional File 2: Table S1), with a single allele (Fig. 1A, D) and a single haplotype (H3) (Fig. 1B, E). Also, the Cameroon and Uganda sampled populations displayed no diversity in *CYP6P4a* (Hd = 0, D = 0, π = 0) and indications of selection of the *CYP6P4b* gene (Additional File 2: Table S1), as most sequences from these regions grouped together with a common major haplotype (H2) (Fig. 1B, D). These haplotypes were fixed in the sampled population in Uganda (CYP6P4a = 10/10, CYP6P4b = 10/10) and close to fixation in the sampled population of Cameroon (CYP6P4a = 12/12, CYP6P4b = 5/7) (Fig. 1C, F). Contrastingly, the insecticide-susceptible FANG population exhibited the highest diversity (Hd = 1 for *CYP6P4a* and Hd = 0.855 for *CYP6P4b*) with no signs of selection, suggesting an association between the selective sweep observed in the four regions and pyrethroid resistance.

### Africa-wide temporal evolution of CYP6P4a and CYP6P4b is associated with increase in pyrethroid resistance

A striking reduction in diversity is observable in both genes in the populations of Uganda, Cameroon, and Ghana between 2014 and 2021 (Fig. 1G, H). In 2014, *CYP6P4a* and *CYP6P4b* were highly diverse, with multiple haplotypes circulating in both permethrin-resistant (AL) and susceptible (DE) mosquitoes from Uganda and Cameroon. In Malawi, however, there was low diversity with the predominance of specific haplotypes selected (Additional File 1: Figs. S1 and S2). Specifically, for *CYP6P4a* in Ghana, the selection was evident through a reduction in haplotype diversity from 0.934 [ from 11 haplotypes (*h*)] in 2014 to 0.583 (*h* = 4) in 2021 with the selection of a predominant allele in the population (Additional File 1: Fig. S2B, Additional File 2: Table S1and S2). An even stronger signature of selection was observed in Cameroon [(2014: *D* = 2.81, *P* < 0.01; Hd = 0.99; *π* = 0.01) vs (2021: *D* = 0, Hd = 0, *π* = 0)] and Uganda [(2014: *D* = 2.25, *P* < 0.05; Hd = 0.78; *π* = 0.01) vs. (2021: *D* = 0; Hd = 0, *π* = 0)] (Additional File 1: Fig. S1A and S2A, Additional File 2: Table S1and S2). In contrast, the Malawian population exhibited low diversity in both 2014 (*D* = − 1.80345; Hd = 0.097; *π* = 0.00076) and 2021 (*D* = 0; Hd = 0.0; *π* = 0), which can be attributed to earlier directional selection observed in the nearby genes *CYP6P9a/b* since 2010 [7] (Additional File 1: Fig. S1A and S2A, Additional File: 2 Tables S1 and S2). The same pattern was observed for *CYP6P4b*, where the genetic diversity was high in 2014 with no indications of directional selection in either Ghana (*D* = 2.24, *P* < 0.05; Hd = 0.917; *π* = 0.009), Uganda (*D* = 2.09, *P* < 0.05; Hd = 0.89; *π* = 0.00592), or Cameroon (*D* = 2.2, *P* < 0.05, Hd = 0.994, *π* = 0.012) (Additional File 1: Figs, S1B and S2C, Additional File 2: Table S2). In contrast, mosquitoes from Malawi exhibited reduced diversity, with the gene undergoing selection (*D* = − 1.84934; Hd = 0.273, *π* = 0.005) (Additional File 1: Fig. S1B and S2C, Additional File 2: Table S2). In 2021, a marked reduction in gene diversity was observed in all populations, with signs of directional selection in Ghana (*D* = − 1.508; Hd = 0.216; *π* = 0.00014) and Cameroon (*D* = − 1.0062; Hd = 0.286; *π* = 0.00019) and no diversity in Uganda (*D* = 0; Hd = 0; *π* = 0) and Malawi (*D* = 0; Hd = 0; *π* = 0) (Additional File 2: Table S1).

### Africa-wide coding sequence polymorphism analysis of CYP6P4a and CYP6P4b

For *CYP6P4a*, the dominant allele in Ghana contained six mutations: *6P4a-I*^*220*^*F*^*221*^*M*^*228*^*F*^*406*^*I*^*407*^*I*^*409*^ hereby also referred to as 6P4a-GHA. The major allele common to Cameroon and Uganda had three mutations: *6P4a- R*^*169*^*E*^*295*^*K*^*297*^*.* In FANG, one of the dominant alleles had three mutations: *6P4a- N*^*286*^*R*^*289*^*S*^*291*^, hereby also referred to as 6P4a-FANG. All the southern populations had a single *6P4a-L*^*488*^ allele circulating in the population (Fig. 1C). Concerning *CYP6P4b*, the dominant allele in Ghana had a single characteristic mutation: *6P4b-E*^*284*^, hereby also referred to as 6P4b-GHA, while the dominant allele common to Uganda and Cameroon had the *6P4b-N*^*288*^ mutation. The FANG allele used for downstream analyses was also identified as *6P4b-T*^*291*^*V*^*294*^*Y*^*399*^, hereby also referred to as 6P4b-FANG (Fig. 1F). Again, the southern Africa populations had the same *6P4b-H*^*26*^ allele. These mutations were mapped to important domains of cytochrome P450s (Additional File 1: Figs. S3A and S3B) and the dominant alleles were used for downstream functional genomic validations.

### Mutations in CYP6P4a increase enzyme’s affinity for pyrethroids

Template multiple sequence alignments (MSA) for Ghana *6P4a-I*^*220*^*F*^*221*^*M*^*228*^*F*^*406*^*I*^*407*^*I*^*409*^ (mutant) and FANG *6P4a- N*^*286*^*R*^*289*^*S*^*291*^ (wild-type) exhibited good sequence depth and coverage, with > 30 template sequences as required for accurate AlphaFold modelling (Additional File 1: Fig. S4E and S4F). All 3D atomic resolution structural coordinates were calculated with high accuracy, reflected in the high average predicted Local Distance Difference Test score (pLDDT > 90%) of the modelled structures (Additional File 1: Figs. S4A and S4B). The pLDDT assesses the prediction reliability for each residue position. Model confidence was further confirmed by the low average predicted aligned error (PAE < 5 Å) which represents the expected distance error between pairs of residues in the predicted structure (Additional File 1: Figs. S4C and S4D). Structural superposition of wild-type and mutant alleles produced a mean backbone accuracy of 0.37 Å r.m.s.d. (Cα root mean square deviation at 95% residue coverage, confidence interval = 0.01– 3.16 Å) (Figs. S4G and S4H). The highest backbone deviation occurred in the *K*^*280*^* – M*^*300*^ helix-loop domain with a mean r.m.s.d. of 1.41 Å, perhaps, strongly indicative of resulting conformational change elicited by three (*M*^*220*^*I*,* V*^*221*^*F*,* V*^*228*^*M*) of the six 6P4a-GHA mutations localised on the third N-terminal helix adjacent to this *K*^*280*^* – M*^*300*^ domain in both models. As a comparison point, the approximate diameter of a carbon atom is 1.5 Å, which is roughly equal to the 1.41 Å r.m.s.d. displacement of the helix-loop. To further estimate (validate) the reliability of the docking algorithms used to approximate the binding affinities between pyrethroids-enzyme alleles, the heme molecule binding mode from the experimentally determined *CYP3A4* template crystal structure was mapped onto 6P4a-GHA and 6P4a-FANG models prior to docking, producing a mean accuracy of 0.35 Å r.m.s.d. (all atom root-mean-square deviation between crystal and computationally predicted heme binding modes; r.m.s.d values < 2 Å represents very confident accuracy) (Additional File 1: Fig. S4G-J).

**Fig. 2:**
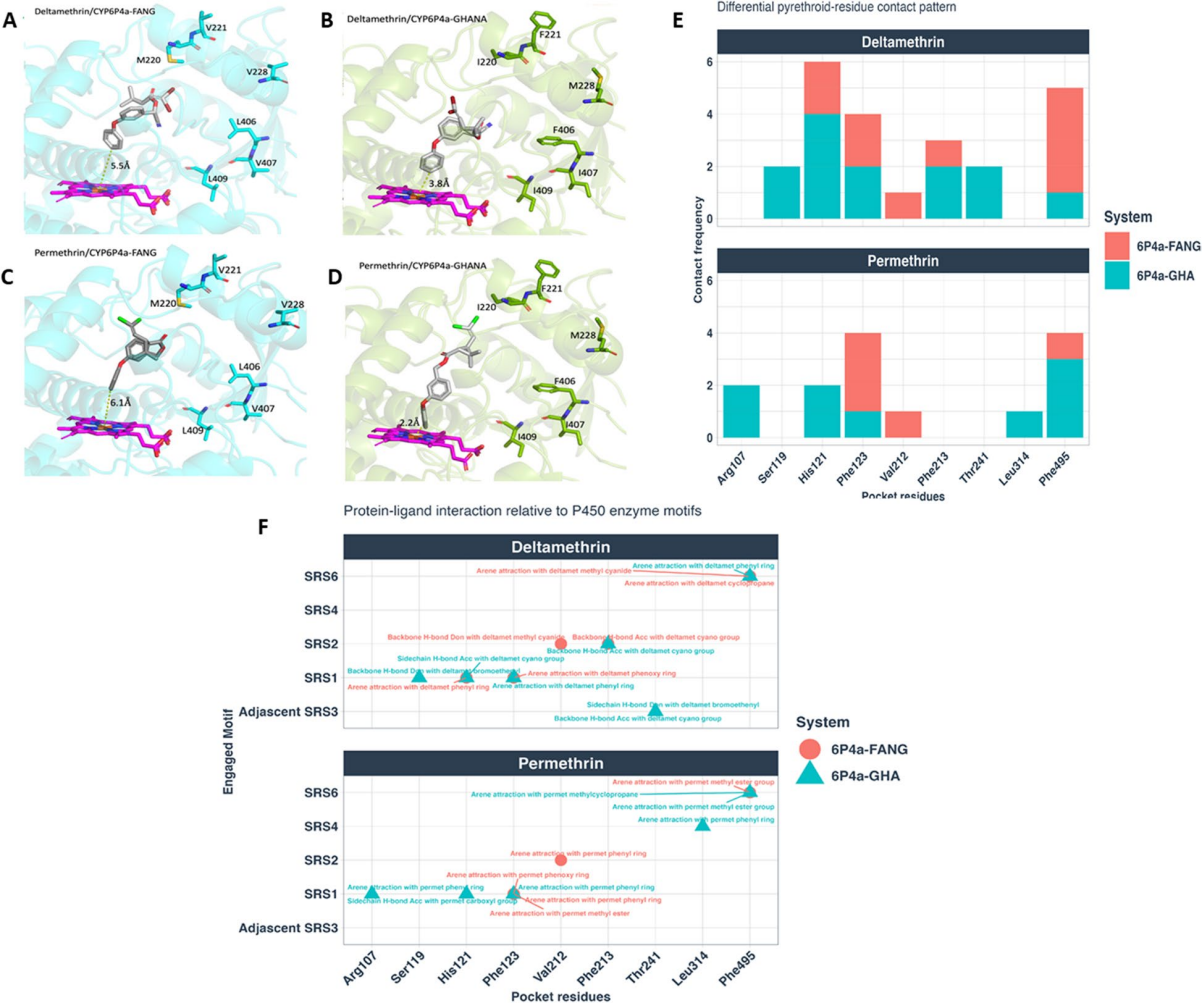
Modelling the CYP6P4a active site and impact of amino acid changes on pyrethroid binding. Predicted 3D binding modes of representative pyrethroid poses with 4′-phenoxy spot approaching above the heme iron at a distance ranging between 1.5 and 6.5Å. Deltamethrin bound to (**A**) CYP6P4a-FANG and (**B**) CYP6P4a-Ghana and permethrin bound to (**C**) CYP6P4a-FANG and (**D**) CYP6P4a-Ghana models. Permethrin and deltamethrin represented in stick format (coloured grey), CYP6P4a-FANG (coloured cyan) and CYP6P4a-Ghana (coloured green) protein models are represented in cartoon with protruded wildtype (CYP6P4a-FANG) vs. mutant (CYP6P4a-Ghana) residues shown as sticks. Heme atoms also in sticks and coloured purple. Distances between possible sites of metabolism and heme iron are annotated in angstrom. Protein–ligand interaction fingerprint between pyrethroid and protein models. **E** Differential pyrethroid-residue contact pattern for deltamethrin (top panel) and permethrin (bottom panel) interacting with both wilt-type and mutant protein models. **F** Interactions portray substrate recognition site (SRS) residues and motifs of wild- and mutant-type proteins interacting with pyrethroids: deltamethrin (first panel) and permethrin (second panel). Comparing the 6P4a-GHA and 6P4a-FANG systems which indicate that more SRS sites were involved in the former. SRS3 and SRS4 motifs exhibited unique intermolecular interactions with deltamethrin and permethrin, respectively. Stronger binding affinity of 6P4a-GHA model was primarily attributed to interactions between deltamethrin and SRS-1, SRS-3, and SRS-6 as well as permethrin and SRS-1, SRS-4, and SRS-6

Following structural modelling, molecular docking was carried out with the validated docking pipeline to assess binding parameters of the pyrethroid ligands (Additional File 1: Fig. S4K) and protein models. Overall, the 3D modelling of CYP6P4a–substrate interactions exhibited good binding scores and limited clashes in the active site. Ligand docking to 6P4a-GHA and 6P4a-FANG revealed contrasting binding conformations and parameter. Docked poses with functional sites approaching above the heme catalytic iron centre within 1.5–6.5 Å to account for optimal van der Waals clashes were shortlisted for downstream analyses. The most enriched pyrethroid binding modes involved the 4′-phenoxybenzyl moiety, than any other moiety, approaching above the heme iron within 1.5–6.5 Å and were thus categorised as productive poses going forward (Fig. 2A–D). Subsequent analyses were performed on productive poses for each pyrethroid bound to 6P4a-FANG or 6P4a-GHA. The differences in the average distances between the 4′-phenoxy spot of all deltamethrin binding modes and the heme iron of the 6P4a-GHA (3.2 ± 0.42 Å) and 6P4a-FANG (4.7 ± 0.38 Å) models as well as permethrin productive poses bound to 6P4a-GHA (3.4 ± 0.6 Å) and 6P4a-FANG (4.3 ± 0.5 Å) models were significant (*P* < 0.018), and both pyrethroids exhibited shorter and more favourable distances for ring hydroxylation at the 4′ position when bound to the 6P4a-GHA model compared to 6P4a-FANG (Fig. 2A–D, Additional File 2: Table S3). Thermodynamic evaluation of affinity of binding for the selected poses predicted that 6P4a-GHA had consistent and significantly (*P* < 0.038) greater affinity for deltamethrin (MM affinity − 9.04 ± 0.11 kcal/mol; GBVI/WSA ΔG − 35.5 ± 0.68 kcal/mol; and S score − 7.4 ± 0.03) than 6P4a-FANG (MM affinity − 8.6 ± 0.13 kcal/mol; GBVI/WSA ΔG − 34.2 ± 1.3 kcal/mol; and S. score − 7.1 ± 0.04) (Additional File 2: Table S3). Likewise, significantly (*P* < 0.029) better binding affinity for permethrin was predicted for the 6P4a-GHA enzyme (MM affinity − 8.85 ± 0.28 kcal/mol; GBVI/WSA ΔG − 34.8 ± 1.2 kcal/mol; S score − 7.1 ± 0.05) compared to 6P4a-FANG (MM affinity − 8.17 ± 0.11 kcal/mol; GBVI/WSA ΔG − 33.5 ± 0.9 kcal/mol; and S score − 6.8 ± 0.05) (Additional File 2: Table S3).

A 250-ns molecular mechanics thermodynamic integration simulation of the interaction between the CYP6P4a models and insecticides confirmed the docking binding affinity approximations, where 6P4a-GHA exhibited better affinity for deltamethrin (mmgb ΔG = − 8.3 kcal/mol) than 6P4a-FANG (mmgb ΔG = − 8.15 kcal/mol) (Additional File 2: Table S3). Affinities for permethrin were also confirmed with 6P4a-GHA binding more strongly to permethrin (mmgb ΔG = − 7.69 kcal/mol) than 6P4a-FANG (mmgb ΔG = − 7.56 kcal/mol) (Additional File 2: Table S3). While these differences in pyrethroid binding free energy between the wild-type and mutant alleles were moderate, the strength of these data lies in the remarkable consistency between the thermodynamic integrations and molecular docking results.

### PLIF reveals possible mechanisms for preferential pyrethroid binding to 6P4a-GHA model

Analyses of protein–ligand interaction fingerprints (PLIF) allowed the identification of key substrate recognition site (SRS) residues and ligand pharmacophores responsible for the preferential binding of pyrethroids to the 6P4a-GHA mutant compared to the wild-type model (6P4a-FANG). Mutations in the 6P4a-GHA model (*M*^*220*^*I*,* V*^*221*^*F*,* V*^*228*^*M*,* L*^*406*^*F*,* V*^*407*^*I*, and *L*^*409*^*I*) induced changes in the active site architecture within SRS regions, leading to decreased efficacy of pyrethroid insecticides. The 6P4a-GHA system had more engaged SRS sites (SRS1–4 and SRS-6) than 6P4a-FANG, with SRS3 and SRS4 motifs forming interactions with deltamethrin and permethrin, respectively (Figs. 2E, F). Stronger binding affinity in the 6P4a-GHA model was predicted to be primarily induced by deltamethrin interactions with hotspot residues SRS-1 (*S*^*119*^,* H*^*121*^), SRS-3 (*T*^*241*^), and SRS-6 (*F*^*495*^) as well as permethrin bonding with SRS-1 (*R*^*107*^,* H*^*121*^), SRS-4 (*L*^*314*^), and SRS-6 (*F*^*495*^) (Figs. 2E and F). Deltamethrin’s bromoethenyl group forms a backbone hydrogen bond with *S*^*119*^, along with arene-H hydrophobic interactions between deltamethrin phenyl-ring and *H*^*121*^ of SRS-1 motif (Figs. 2E, F). For permethrin, the key interactions within SRS-1 predicted to influence the preference of the mutant 6P4a-GHA system are the arene attraction between *R*^*107*^ and permethrin’s phenyl ring and *H*^*121*^ sidechain hydrogen bond donation to permethrin’s carboxylic-ester group (Figs. 2E, F). Structural changes induced by the 6P4a-GHA model mutations in the SRS-6 region, particularly at residue *F*^*495*^, enhanced substrate selectivity for permethrin. *F*^*495*^ formed arene-H interactions with methyl-ester and dimethyl-cyclopropane groups of permethrin, contributing to its binding affinity in the mutant enzyme. Furthermore, the 2-D interaction maps reveal that metabolism of deltamethrin by the mutant allele is driven by hydrogen bonding and arene-π electron interaction while binding to permethrin is mainly through arene-arene, arene-H, and arene-cationic hydrophobic interactions (Additional File 1: Fig. S5). This was reflected in the binding affinities, where the mutant allele exhibited more affinity for deltamethrin than for permethrin (Additional File 2: Table S3).

Docking ligands to Ghana *6P4b-*^*E284*^ (6P4b-GHA) and *6P4b-T*^*291*^*V*^*294*^*Y*^*399*^ (6P4b-FANG) produced inconsistent affinity and binding mode differences and was not further investigated.

### CYP6P4a gene is duplicated in Ghana

Recent studies have highlighted gene amplification of P450s as an insecticide resistance response mechanism. Here, gene duplication of *CYP6P4a* was confirmed through copy number variation study using qPCR (Additional File 1: Fig. S6). A twofold copy number of *CYP6P4a* was observed in the Ghanian *An. funestus* compared to the susceptible FANG laboratory. Quantification of copy number for *CYP6P4b* was challenging due to the non-specificity of the primers to amplify the Ghanian samples.

### Recombinant An. funestus CYP6P4a and CYP6P4b enzymes metabolise pyrethroids insecticides

**Fig. 3:**
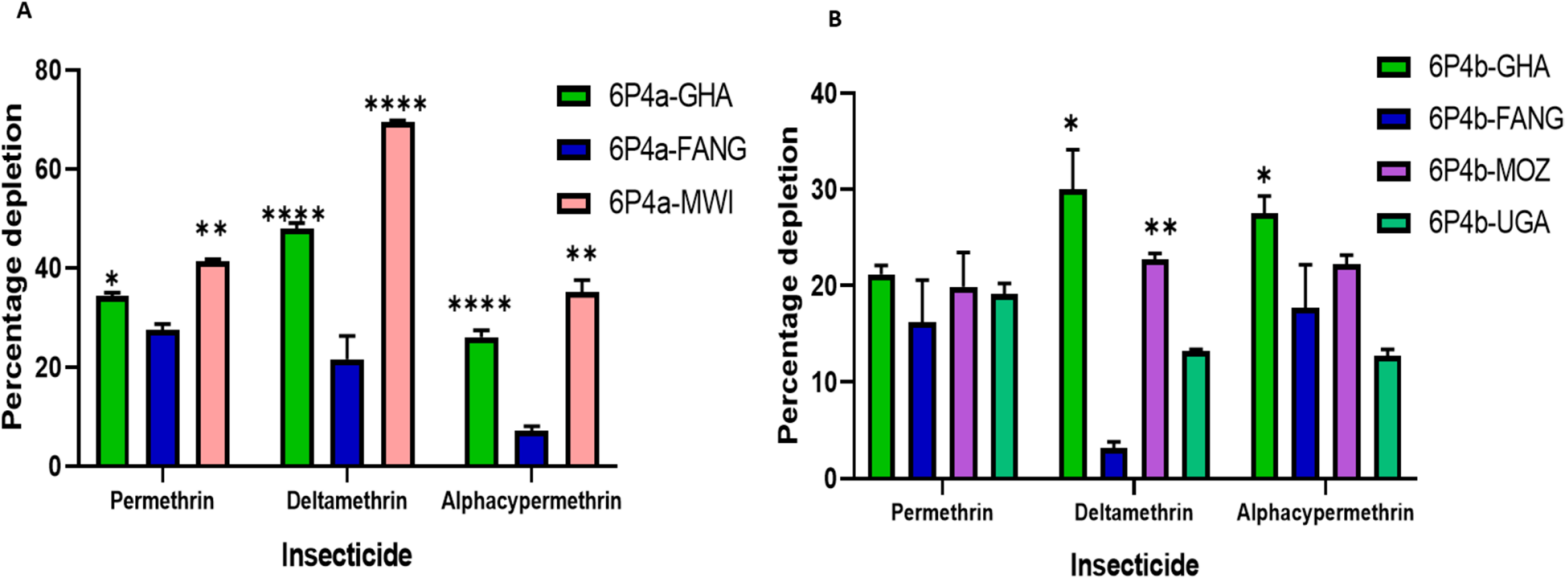
In vitro assessment of pyrethroid metabolism by CYP6P4a and CYP6P4b candidate alleles. **A** CYP6P4a recombinant enzyme metabolism of pyrethroids. **B** CYP6P4b recombinant enzyme metabolism of pyrethroids. Values are mean ± SEM of three experimental replicates compared with negative control without NADPH. Depletion by recombinant resistant enzymes is significantly different from depletion by FANG recombinant enzymes at *P < 0.05, **P < 0.01, ***P < 0.001, and ****P < 0.0001

Optimal expression of the enzymes was achieved 22 h post-induction with IPTG (Additional File 1: Fig. S7A). Substrate disappearance assays (Additional File 1: Fig. S7B) revealed that the Ghanaian *6P4a-I*^*220*^*F*^*221*^*M*^*228*^*F*^*406*^*I*^*407*^*I*^*409*^ (6P4a-GHA) and Malawian *6P4a-L*^*488*^ (6P4a-MWI) enzymes significantly metabolised permethrin (*P* < 0.05 and *P* < 0.01, respectively), with 34.34% and 41.33% depletion respectively, compared to 6P4a-FANG with 27.57% depletion of the insecticide (Fig. 3A). For type II pyrethroids, 6P4a-GHA and 6P4a-MWI demonstrated significantly higher depletion of deltamethrin, depleting 47.88% (*P* < 0.0001) and 69.59% (*P* < 0.0001), respectively, compared to 21.59% depletion by the 6P4a-FANG. This corresponds to 2.2-fold and 3.2-fold, respectively, of elevated metabolic activity by the resistance alleles. Similarly, for alphacypermethrin, 6P4a-GHA and 6P4a-MWI significantly depleted the insecticide by 26.04% (3.7-fold) and 35.13% (4.9-fold) respectively, compared to the FANG variant that exhibited minimal metabolism (7.1%) (Fig. 3A). Similar patterns were observed for CYP6P4b enzymes, although to a lesser extent. For permethrin, the Ghanaian *6P4b-E*^*284*^ (6P4b-GHA), the Mozambican *6P4b-H*^*26*^ (6P4b-MOZ), and Ugandan *6P4b-N*^*288*^ (6P4b-UGA) exhibited depletions of 21.20%, 20.0%, and 19.11%, respectively, with no significant difference when compared to FANG *6P4b-T*^*291*^*V*^*294*^*Y*^*399*^ (6P4b-FANG) with 16.22% depletion (Fig. 3B). With deltamethrin, 6P4b-GHA demonstrated a significant depletion of 30.05% (*P* = 0.001), 6P4b-MOZ exhibited 22.73% depletion (*P* = 0.0004), the 6P4b-UGA showed 13.30% depletion, while 6P4b-FANG exhibited little or no metabolism of the insecticide with only 3.15% depletion. This corresponds to an approximate tenfold, 7.2-fold, and 4.2-fold elevated metabolic activity when compared to the FANG variant. The enzymes also metabolised alphacypermethrin, with 6P4b-GHA, 6P4b-MOZ, 6P4b-UGA, and 6P4b-FANG producing 27.5%, 22.2%, 12.8%, and 17.7% depletion, respectively, with a significant difference obtained for the 6P4b-GHA vs. 6P4b-FANG comparison (*P* < 0.04) (Fig. 3B).

### In vivo*, *allelic variation and overexpression of CYP6P4a and CYP6P4b confer insecticide resistance

**Fig. 4:**
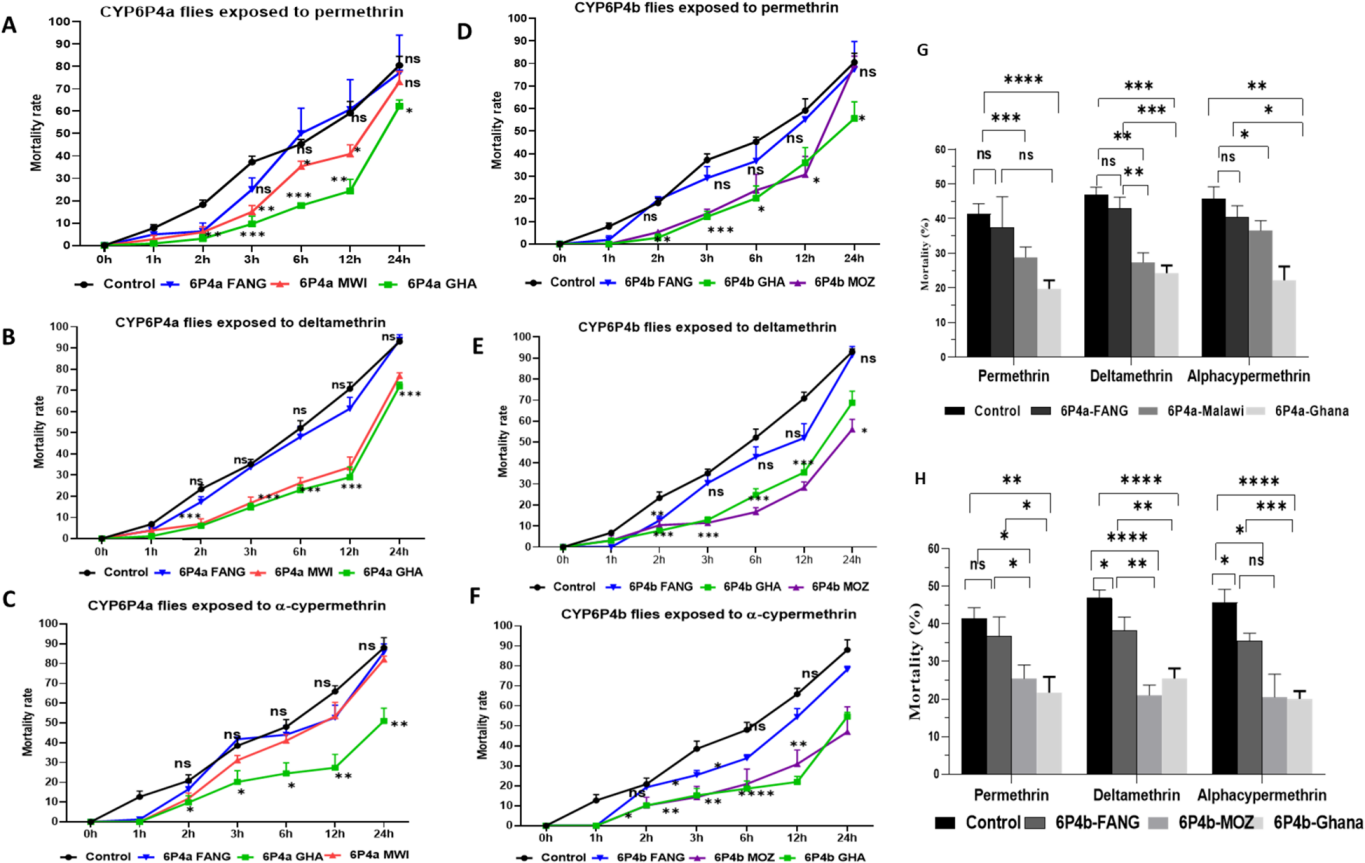
Twenty-four-hour insecticide contact bioassays with transgenic flies over-expressing *CYP6P4a* alleles exposed to (**A**) permethrin, **B** deltamethrin, and (**C**) alphacypermethrin and transgenic flies over-expressing *CYP6P4b* alleles exposed to (**D**) permethrin, **E** deltamethrin, and (**F**) alphacypermethrin. Data shown is mean ± S.E.M; significant difference in mortality is indicated for the GHA vs control and FANG vs control comparisons. Cumulative mortalities of transgenic flies and control flies to insecticides for all six time points. **G** For CYP6P4a transgenic fly experiment. **H** For CYP6P4b transgenic fly experiment. *P < 0.05, **P < 0.01, ***P < 0.001, and ****P < 0.0001

Given the overexpression of *CYP6P4a* and *CYP6P4b* in field populations, transgenic *Drosophila* flies were constructed to assess the impact of the independent and sole over-expression of each of the genes on resistance to pyrethroids and, further, to investigate the impact of allelic variation on resistance in an in vivo system. Quantitative RT-PCR analysis confirmed the significant overabundance of *CYP6P4a* and *CYP6P4b* transcripts in the transgenic *D. melanogaster* compared to non-transgenic control strains (Additional File 1: Fig. S8). Throughout 24h exposure to permethrin, GAL4-CYP6P4a-GHA flies and GAL4-CYP6P4a-MWI showed significantly lower mortality rates at different time intervals compared to the GAL4-CYP6P4a-FANG flies and the non-transgenic group (Fig. 4A). Also, averagely, a significant higher resistance was observed in the experimental group compared to the control group and GAL4-CYP6P4a-FANG flies throughout the 24h while revealing no difference in mean mortality between GAL4-CYP6P4a-FANG and the control group (Fig. 4G). This implies that at any given time of exposure, the experimental group was more resistant than the non-transgenic group, and the overexpression of the GAL4-CYP6P4a-FANG allele confers no significant resistance to permethrin (Fig. 4G). Similarly, GAL4-CYP6P4a-GHA and GAL4-CYP6P4a-MWI flies were significantly more resistant to deltamethrin than GAL4-CYP6P4a-FANG and the control group at all time points (Fig. 4B). Notably, after 12 h of deltamethrin exposure, GAL4-CYP6P4a-GHA and GAL4-CYP6P4a-MWI flies recorded 29% and 33.67% mortality, while GAL4-CYP6P4a-FANG and the control exhibited 61% and 70% mortality, respectively (Fig. 4B). Data also revealed a significantly high resistance in the experimental group compared to the control group and GAL4-CYP6P4a-FANG flies at all six different times, spanning 1 h–24 h, while no difference was recorded between the control group and the GAL4-CYP6P4a-FANG flies (Fig. 4G). For alphacypermethrin, the GAL4-CYP6P4a-GHA flies exhibited significant resistance to the insecticide compared to the GAL4-CYP6P4a-FANG and control groups, but overexpression of the MWI allele conferred significantly more resistance to alphacypermethrin compared to the control group only after 1h exposure (Fig. 4C). However, data showed a significantly high resistance in the GAL4-CYP6P4a-MWI compared to the control group when comparing mean mortalities for all six time points (36.59% vs. 45.66%) (Fig. 4G). Similarly, GAL4-CYP6P4b-GHA and GAL4-CYP6P4b-MOZ transgenic lines exhibited more resistance compared to GAL4-CYP6P4b-FANG and control flies. After 12 h of permethrin exposure, GAL4-CYP6P4b-GHA and GAL4-CYP6P4b-MOZ flies exhibited significantly lower mortalities (36.05% and 30.73%, respectively) than the GAL4-CYP6P4b-FANG and control flies (44.35% and 59.17% respectively) (Fig. 4D). Mean mortalities for all six time points were 21.73%, 25.45%, 30.70%, and 41.38% for GAL4-CYP6P4b-GHA, GAL4-CYP6P4b-MOZ, GAL4-CYP6P4b-FANG flies, and the non-transgenic group, respectively (Fig. 4H). With deltamethrin, GAL4-CYP6P4b-GHA and GAL4-CYP6P4b-MOZ transgenic lines displayed lower mortality rates (35.56% and 28.30%, respectively) after 12 h of exposure, while GAL4-CYP6P4b-FANG and the control recorded 51.93% and 70.74% mortality, respectively (Fig. 4E). For alphacypermethrin, GAL4-CYP6P4b-GHA and GAL4-CYP6P4b-MOZ exhibited higher resistance with mortalities of approximately 21.96% and 30.83%, respectively, compared to the control group with mortalities of 54.5% and 65.9% after 12 h of exposure (Fig. 4F). Average mortalities for all six time points indicated that all transgenic groups were more resistant than the control group except for GAL4-CYP6P4b-FANG flies’ exposure to permethrin (Fig. 4H). These results collectively indicate that the upregulation of *CYP6P4a* and *CYP6P4b* confers resistance to pyrethroids but with allelic variation being a key factor in the phenotype as overexpression of the FANG variants did not confer resistance.

### Two new molecular markers to track CYP6P4a and CYP6P4b-mediated pyrethroid resistance

**Fig. 5:**
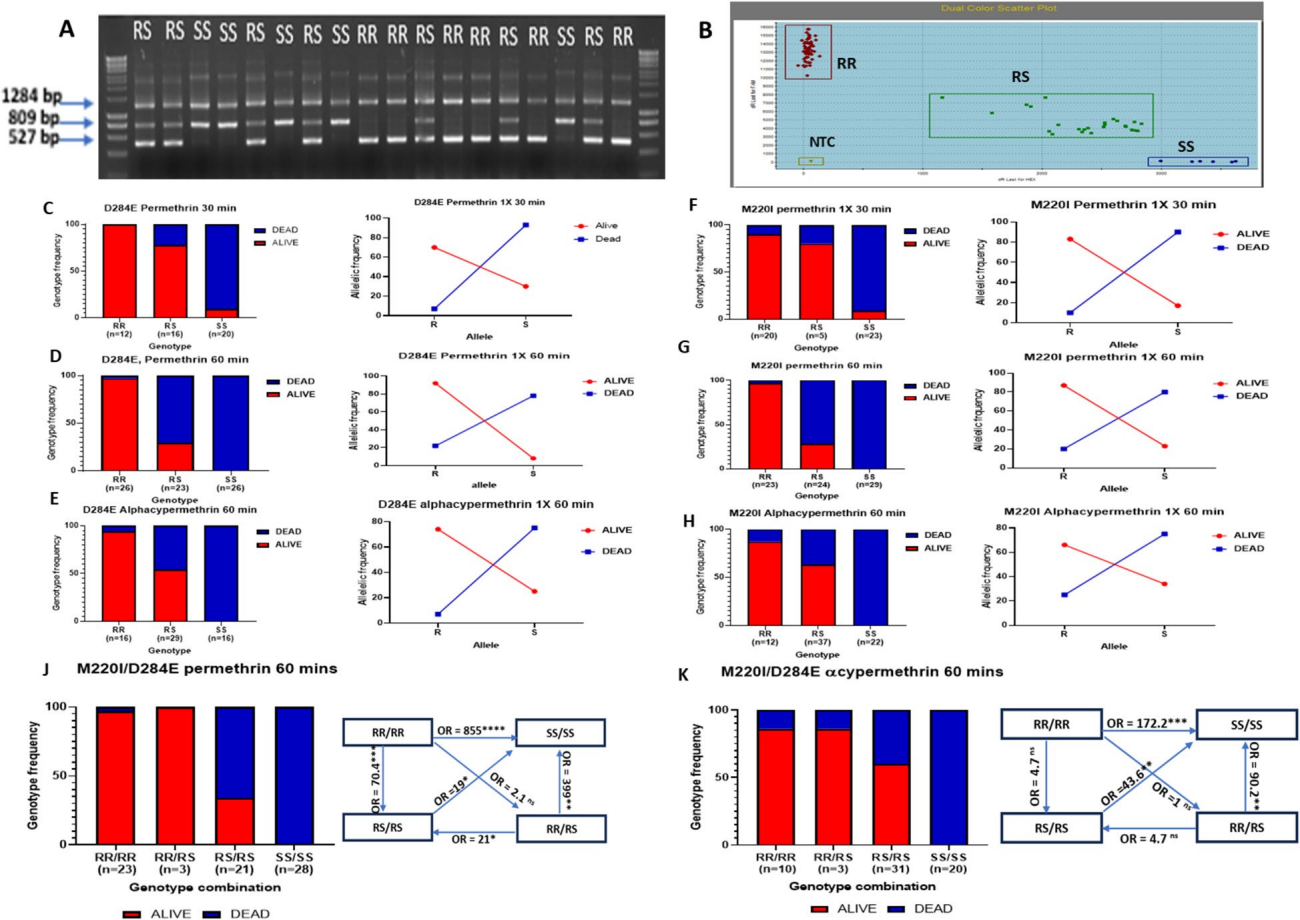
Design of DNA-based molecular diagnostic tools for detection of *CYP6P4a* and *CYP6P4b* resistance alleles and impact on resistance phenotype*.* **A** ARMS-PCR assay for genotyping the CYP6P4b-D284E marker. **B** LNA probe-based assay for genotyping the CYP6P4a-M220I marker. Association of the CYP6P4b-D284E and the CYP6P4a-M220I mutations with insecticide resistance phenotype. Distribution of CYP6P4b-D284E resistance marker among F3 FANGxGHANA hybrid *An. funestus* mosquitoes exposed to (**C**) permethrin for 30 min, **D** permethrin for 60 min, and (**E**) alphacypermethrin for 60 min. Distribution of CYP6P4a-M220I resistance marker among F3 FANGxGHANA hybrid *An. funestus* mosquitoes exposed to (**F**) permethrin for 30 min, **G** permethrin for 60 min, and (**H**) alphacypermethrin for 60 min. 284E, 220I (R) and D284, M220 (S) allele frequency distributions between alive and dead mosquitoes are shown in line plots. Combined impact of CYP6P4b-D284E and CYP6P4a-M220I mutations on pyrethroid insecticide resistance. Distribution of genotypes in FANGxGHANA hybrid *An. funestus* mosquitoes exposed to (**J**) permethrin for 60 min and (**K**) alphacypermethrin for 60 min with odds ratio calculations to compare the survival capacity of double homozygote-resistant mosquitoes after pyrethroid exposure with other genotype combinations

Polymorphism analyses of full-length cDNA sequences allowed the identification of key mutations that were uniquely found in the Ghanaian population and absent in FANG. For *CYP6P4b*, the C to A nucleotide change at position 852 led to amino acid change from aspartic acid (GAC) to glutamic acid (GAA) at position 284 (Additional File 1: Fig. S9A). This mutation was used to design a DNA-based diagnostic tool (CYP6P4b-D284E) following the Amplification Refractory Mutation System (ARMS) PCR technique (Fig. 5A). For *CYP6P4a*, six amino acid changes were identified: M220I, V221F, V228M, L406F, V407I, and L409I (Additional File 1: Fig. S9B). Among these, the methionine-to-isoleucine change at position 220 (M220I) was selected due to its proximity to substrate recognition site 2 (SRS-2) (Additional File 1: Fig. S2A) and its role in the binding pocket of the enzyme, as revealed by molecular docking simulations. The proximity of M220I, V221F, and V228M mutations led to the formation of a region containing numerous SNPs complicating the development of a diagnostic assay for *CYP6P4a* targeting 1 mutation as for *CYP6P4b*. To address this, a more precise PCR diagnostic tool assay (CYP6P4a-M220I) was designed utilising a locked nucleic acid (LNA) probe targeting this specific mutation haplotype (Fig. 5B). The detailed protocols for the assay can be found in Additional File 2: Tables S4 and S5.

### CYP6P4a and CYP6P4b markers are strongly associated with pyrethroid insecticide resistance

Genotyping the CYP6P4a-M220I marker in the field mosquitoes in Ghana revealed that 69.5% of individuals (32/46) were homozygous 220I/220I (RR), 21.74% (10/46) were heterozygous M220/220I (RS), and 8.7% (4/46) were homozygous susceptible M220/M220 (SS) (Additional File 1: Fig. S9C). This corresponds to an allelic frequency distribution of 79.2% and 20.8% for the R and S alleles in the population. For the CYP6P4b-D284E marker, a higher percentage of the resistance allele was recorded: 95.65% (44/46) 284E/284E (RR) and 4.35% (2/46) D284/284E (RS) genotypes, with no D284/D284 (SS) individual. This corresponds to a 97.8% frequency of the R allele and 2.2% frequency of the S allele in the population (Additional File 1: Fig. S9C). In contrast, all forty FANG samples were homozygous SS for both markers confirming the efficacy of the diagnostic tool. The high pyrethroid resistance and high frequency of the resistant alleles in the field population impeded the establishment of a genotype–phenotype correlation. We opted for a genetic cross strain between the highly resistant Ghanaian mosquitoes and the fully susceptible laboratory strain FANG to establish a population of mosquitoes with all three genotypes to allow genotype/phenotype correlation studies. At the F3 generation, the population was made of 25% RR, 35% RS, and 40% SS individuals for the CYP6P4b-D284E marker (Additional File 1: Fig. S9D), confirming that the genetic cross successfully segregated the genotypes. These mosquitoes were exposed to pyrethroids using the WHO insecticide bioassays for the establishment of the genotype–phenotype association (Additional File 1: Fig. S10A and S10B). Exposing mosquitoes to permethrin 1X for 30 min resulted in 61% mortality, while after 1 h, mortality was 85 ± 2.57% for permethrin 1X and 54 ± 2.57% for alphacypermethrin 1X (Additional File 1: Fig. S10A). These mortalities are far higher than the 11.6 ± 5% for permethrin 1X and 1.25 ± 1.25% for alpha-cypermethrin 1X, previously recorded in the field mosquitoes in the same year [15], highlighting the potential association between these markers and the phenotype.

#### CYP6P4b-D284E

Eighty-six percent of highly susceptible mosquitoes (dead after 30-min exposure to permethrin) were homozygous susceptible SS (18/21) and 14% heterozygotes (3/21) with no homozygous resistant RR individuals observed (Fig. 5C left). In contrast, highly resistant mosquitoes that survived 60-min exposure to permethrin were predominantly homozygous RR (25/30), and heterozygotes (5/30), constituting 83% and 17% of the highly resistant mosquitoes respectively, with no SS individual (Fig. 5D left). Allele frequency distribution further supported the trend where in highly resistant mosquitoes, 97% R and 20% S allele frequencies were recorded, while in the highly susceptible mosquitoes, the R allele was present at 7% and the S allele was present at 93% (Fig. 5C right and 5D right). A strong positive association was established between CYP6P4b-D284E marker and ability to survive exposure to permethrin, with RR individuals having greater chance of surviving compared to SS individuals (OR = 901; CI = 35.06 to 23,156.34; *P* < 0.0001, Fisher’s exact test) (Additional File 2: Table S6). Similar observations were made for mosquitoes exposed to alphacypermethrin for 60 min, where carrying the RR genotype enhanced alphacypermethrin resistance, reflected in the significant odds ratios observed in the RR vs. SS comparison (OR = 341; CI = 12.09 to 9016.19; *P* < 0.0001, Fisher’s exact test) and RS vs. SS comparison (OR = 40.33; CI = 2.21 to 735.99; *P* = 0.0126, Fisher’s exact test) (Fig. 5E left). These findings were further supported by the allele frequency distribution, with the R allele occurring at 74.2% in surviving mosquitoes and 25.8% in the dead mosquitoes, while the S allele frequency was 25% in the surviving mosquitoes and 75% in the dead mosquitoes (Fig. 5E right). Overall, for resistance to both type 1 and 2 pyrethroids, an additive effect was observed as RR individuals survived significantly more than RS individuals (Additional File 2: Table S6).

#### CYP6P4a-M220I

Eighty-eight percent of the highly susceptible mosquitoes (dead after 30-min exposure to permethrin) were SS (21/24), 4% RS (1/24), and 8% RR (2/24) (Fig. 5F left). In contrast, the mosquitoes that survived after 60 min exposure to permethrin were predominantly RR (22/30) and RS (8/30), with no SS individual (Fig. 5G left). Furthermore, the R allele was more prevalent among the survivors (87%), while the S allele was more common among the dead mosquitoes (80%) (Fig. 5G right). These findings established a strong association between the CYP6P4a-M220I marker and permethrin resistance with highly significant odds ratio when comparing RR vs. SS (OR = 885; CI = 34.41 to 22,763.86; *P* < 0.0001), Fisher’s exact test) and RS vs. SS (OR = 30.4; CI = 1.65 to 560.83; *P* = 0.0217, Fisher’s exact test). Similar results were obtained with alphacypermethrin as resistant mosquitoes were predominantly RR (10/31) and RS (21/31), with no SS individual (0/30) while the dead were predominantly SS (22/40) and RS (16/40), with 2 RR individuals (2/40). Significant odds ratios were observed for the RR vs. SS (OR = 189; CI = 8.32 to 4295.30; *P* = 0.001, Fisher’s exact test) and RS vs. SS (OR = 58.64; CI = 3.81 to 1039.32; *P* = 0.0055, Fisher’s exact test) comparisons (Fig. 5H left). This was supported by allele frequency distributions, with the R allele occurring at 66% in the alive mosquitoes and 75% S allele in the dead (Fig. 5H right).

#### CYP6P4a-220I and CYP6P4b-284E combined to confer higher resistance levels

Double homozygote resistant genotype (RR/RR) conferred significantly more resistance to permethrin while double heterozygous genotype (RS/RS) conferred resistance but to a lower extent. For type II pyrethroid (alphacypermethrin), RR/RR and RS/RS genotypes conferred similar resistance levels (Additional File 2: Table S6). Specifically, individuals that survived permethrin exposure were more of the RR/RR (22/30) accounting for 73.3%, RR/RS (3/30) representing 10% and RS/RS (5/30) representing 16.7% of the alive mosquitoes. The majority of individuals dead after exposure were SS/SS (28/45) making up 62.2% and RS/RS (16/45) with only one RR/RR individual (2.2%) (Fig. 5J). A strong genotype phenotype correlation was therefore established in the RR/RR vs SS/SS comparison (OR = 855; *P* < 0.0001, Fisher’s exact test) as well as the other genotype combinations (Additional File 2: Table S6). The additive effect of the markers was also observed with RR/RR individuals surviving significantly more than RS/RS individuals (OR = 70.4; CI = 7.48 to 662.33; *P* = 0.0002) (Fig. 5J right, Additional File 2: Table S6). For alphacypermethrin, all alive individuals had at least one resistant allele, while the dead were more of the double homozygous susceptible genotype (Fig. 5K). Having both CYP6P4a-220I and CYP6P4b-284E resistance alleles in the RR/RR and RS/RS states significantly increased resistance to alphacypermethrin compared to the double homozygote susceptible (SS/SS) state (OR = 172.2; CI = 7.56 to 3923.94; *P* = 0.0012 and OR = 43.6; CI = 2.43 to 785.21; *P* = 0.0104, respectively). The analysis of resistance to alphacypermethrin revealed that the impact of RR/RR genotype on resistance was not significantly different from other genotype combinations. This suggests that individuals do not necessarily require the RR/RR genotype to exhibit high resistance to alphacypermethrin (Fig. 5K right, Additional File 2: Table S6).

### CYP6P4a and CYP6P4b resistance alleles reduce the efficacy of pyrethroid-based tools

Cone assays performed with different LLINs using F4 FANG/GHANA mosquitoes revealed mortalities rate of 30.45 ± 4.94%, 44.09 ± 6.57%, and 32.44 ± 5.74% respectively with PermaNet 2.0 (deltamethrin), DuraNet (alphacypermethrin), and Olyset (permethrin) (Additional File 1: Fig. S10B).

#### CYP6P4b-D284E

**Fig. 6:**
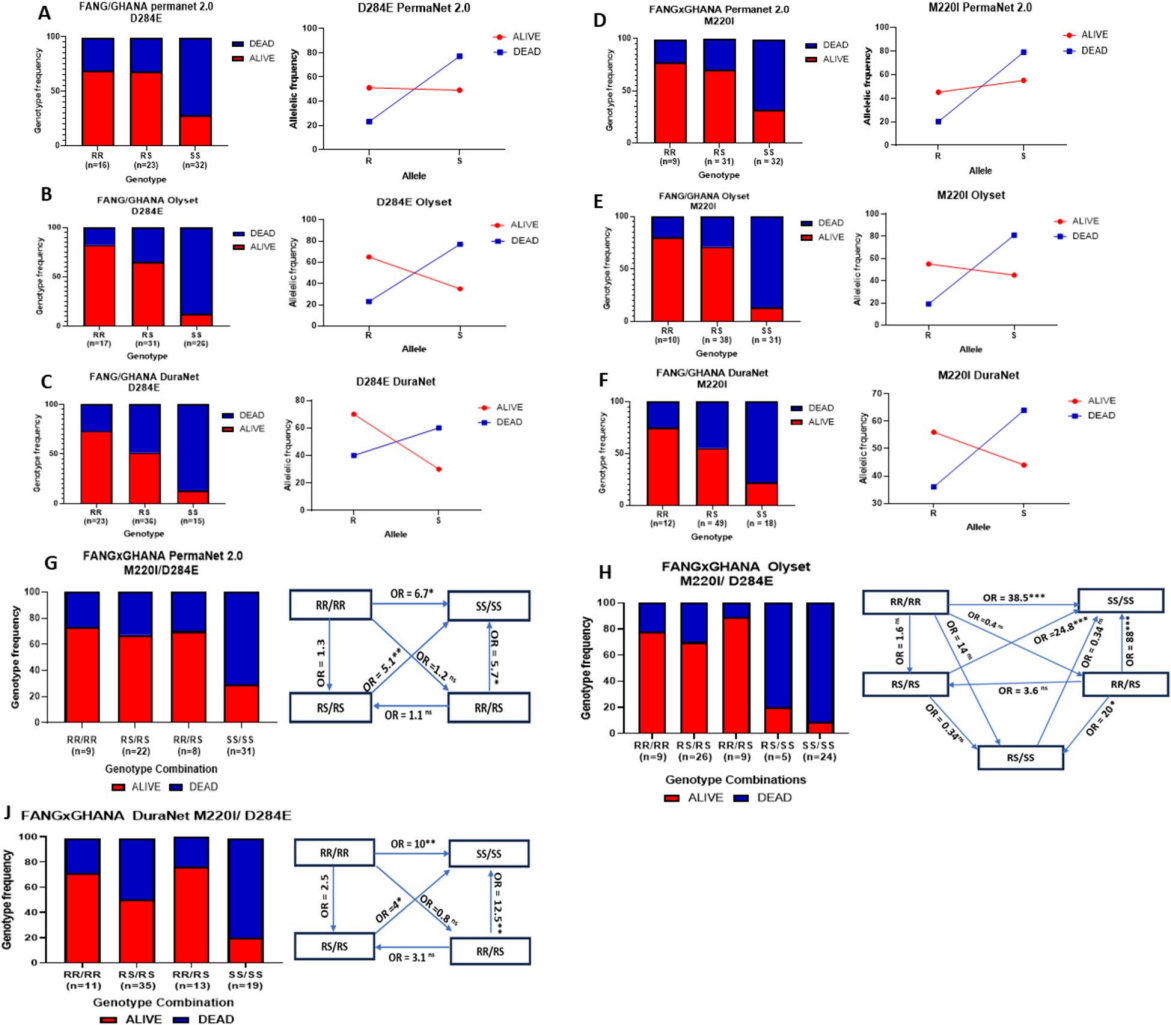
Genotype/phenotype association of the CYP6P4b-D284E and the CYP6P4a-M220I mutations with pyrethroid bed net efficacy. Distribution of CYP6P4b-D284E resistance mutation genotypes among resistant and susceptible F4 FANGxGHANA hybrid *An. funestus* mosquitoes exposed to (**A**) PermaNet 2.0 for 3 min, **B** Olyset for 3 min, and (**C**) DuraNet for 3 min. Distribution of CYP6P4a-M220I resistance mutation genotypes among resistant and susceptible F4 FANGxGHANA hybrid *An. funestus* mosquitoes exposed to (**D**) PermaNet 2.0 for 3 min, **E** Olyset for 3 min, and **F** DuraNet for 3 min. R and S allele frequency distributions between alive and dead mosquitoes after exposure are shown in line plots. Combined impact of CYP6P4b-D284E and CYP6P4a-M220I mutations on pyrethroid bed net efficacy. Distribution of genotypes in FANGxGHANA hybrid *An. funestus* mosquitoes exposed to (**G**) PermaNet 2.0 for 3 min, **H** Olyset for 3 min, and (**J**) DuraNet for 3 min with odds ratio calculations to compare the survival capacity of double homozygote-resistant mosquitoes after pyrethroid exposure with other genotype combinations

Assessing the correlation between genotypes and survivorship revealed that RR mosquitoes were significantly more able to survive exposure to PermaNet 2.0 net than SS mosquitoes (OR = 5.8; CI = 1.49 to 22.01; *P* < 0.0001) (Fig. 6A). A similar observation was made for RS mosquitoes which survived more than SS (OR = 5.4; CI = 1.6579 to 17.6481; *P* = 0.0051). Although mosquitoes that survive had a higher frequency of RR than RS genotype, the difference was not significant (OR = 2.6; CI = 0.6034 to 10.9184; *P* = 0.202) (Additional File 2: Table S7) confirming the pattern observed for WHO tube test with alphacypermethrin. The impact was even greater on Olyset efficacy (Fig. 6B) where homozygous RR and heterozygous RS individuals significantly survived better than homozygous SS individuals (OR 35.8; CI = 6.33 to 202.37; *P* < 0.0001 and OR 14.0; CI = 3.40 to 57.11; *P* = 0.0003). Analogous observations were made for DuraNet with RR and RS individuals exhibiting more resistance than SS individuals (Additional File 2: Table S7, Fig. 6C).

#### CYP6P4a-M220I

Genotyping alive and dead mosquitoes after exposure to PermaNet 2.0 also revealed that CYP6P4a-220I homozygous resistant mosquitoes (RR) were significantly more able to survive exposure to PermaNet 2.0 nets than the homozygous susceptible CYP6P4a-M220 mosquitoes (SS) (OR = 6.7; CI = 1.36 to 34.35; *P* = 0.0205). Similarly, heterozygote mosquitoes survived better than susceptible SS (OR = 4.67; CI = 1.66 to 14.50; *P* = 0.0054) (Fig. 6D). Also, RR and RS genotypes significantly increased chances of surviving exposure to Olyset compared to SS genotype (OR = 27; *P* < 0.0001; and OR = 16.57; *P* < 0.0001) (Fig. 6E). Similarly, double homozygous RR and heterozygous mosquitoes survived more after coming in contact with DuraNet than the homozygous SS mosquitoes (OR = 10.5; *P* = 0.01 and OR = 4.295; *P* < 0.05, respectively) (Fig. 6F).

#### Combined effect of both markers on pyrethroid bed net efficacy

An independent segregation of genotypes at both genes was observed at F4, with several genotype combinations obtained, including RR/RR, RR/RS, RS/RS, RS/SS, and SS/SS. Genotype/phenotype comparisons revealed that RR/RR and RS/RS mosquitoes survived exposure to PermaNet 2.0 significantly more than SS/SS (OR = 6.7; CI = 1.18 to 37.79; *P* = 0.032 and OR = 5.1; CI = 1.55 to 16.71; *P* = 0.0073, respectively) (Fig. 6G). With Olyset, RR/RR, RR/RS, and RS/RS individuals had far greater ability to survive relative to SS/SS individuals (OR = 38.5; *P* = 0.0008, OR = 24.8; *P* = 0.0002, OR = 88; *P* = 0.0005, respectively) and (Fig. 6H, Additional File 2: Table S7). A similar pattern was observed for exposure to DuraNet, where RR/RR, RR/RS, and RS/RS individuals also survived significantly more than SS/SS individuals (OR = 10; CI = 1.78 to 56.15; *P* = 0.009; OR = 12.5; CI = 2.29 to 68.25; *P* = 0.0035, OR = 4; CI = 1.10 to 14.38; *P* = 0.036, respectively) (Fig. 6J).

### Africa-wide distribution of CYP6P4a and CYP6P4b resistance alleles

**Fig. 7:**
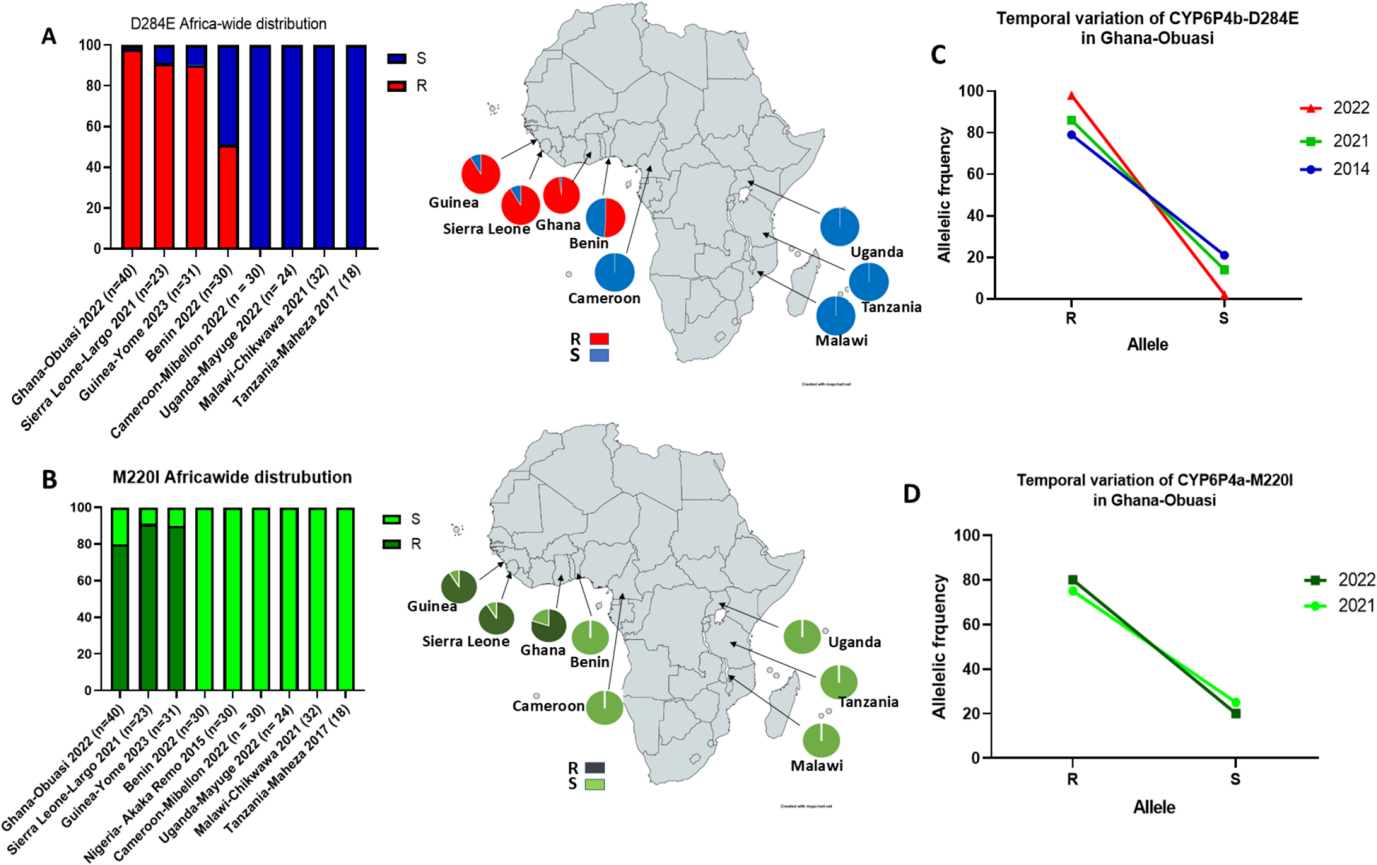
Geographical distribution of molecular markers across Africa. **A** CYP6P4b-D284E resistance allele, **B** CYP6P4a-M220I resistance allele. **C** Temporal assessment of the allele frequency distribution of the CYP6P4b-D284E resistance marker in the *An. funestus* population of Ghana-Obuasi. **D** Temporal assessment of the allele frequency distribution of the CYP6P4a-M220I resistance marker in the *An. funestus* population of Ghana-Obuasi

Screening countries across different regions of Africa, including West, East, Central, and South Africa for the presence of the CYP6P4b-D284E and CYP6P4a-M220I resistance markers, partially established the geographical spread of the markers on the continent. The CYP6P4b-284E resistance marker was close to fixation in Ghana (98%), Sierra Leone (91%), and Guinea (90%) at an intermediate frequency in Benin (51%), all West African countries, but absent in other countries including Cameroon and the DRC in Central Africa, Uganda and Tanzania in East Africa, and Mozambique and Malawi in Southern Africa (Fig. 7A). Also, the CYP6P4a-220I resistance marker was also identified in Ghana, Seirra Leone, and Guinea at high frequencies (80%, 91%, and 90% respectively) and absent in the other afore-mentioned countries (Fig. 7B). Assessment of the evolution of the markers between 2014 and 2022 in the sampled Ghana population revealed that the CYP6P4b-284E marker has increased significantly in the population from 79% in 2014 to 98% in 2022 (*P* < 0.0001), while the CYP6P4a-220I marker has increased although not significantly from 75% in 2014 to 80% in 2022, with chances of getting fixed in the population in a few generations (Fig. 7C, D).

## Discussion

In this study, we elucidated the genetic basis of metabolic resistance to pyrethroids in West African populations of the major malaria vector *An. funestus* establishing that *CYP6P4a* and *CYP6P4b* are driving pyrethroid resistance through overexpression and allelic variation. Furthermore, we introduce the two field-applicable DNA-based diagnostic markers for detecting P450-based metabolic resistance to pyrethroids in *An. funestus* populations in West Africa to facilitate the detection, monitoring, and management of insecticide resistance.

### Directional selection of CYP6P4a and CYP6P4b is associated with pyrethroid resistance

Capturing the evolutionary pattern of the genetic variation of genes is important in the assessment of the impact of insecticide pressure on the population structure. Sequence analyses of mosquitoes collected in 2014 revealed that in most regions, notably in Central (Cameroon) and East (Uganda) Africa, both pyrethroid-resistant (AL) and susceptible (DE) mosquitoes exhibited high diversity for both *CYP6P4a* and *CYP6P4b*, and no selection ongoing at this locus. Analysing the sequences of representative populations of the four regions of Africa in 2021 revealed an overall reduced diversity of *CYP6P4a* and *CYP6P4b* within populations, with the existence of major alleles in each population. The sequences confirmed the presence of four major haplotypes, forming four geographical clusters: southern Africa (Mozambique/Malawi), in East-Central Africa (Uganda/Cameroon), Benin (West), and Ghana alone (West). This population clustering is interestingly very close to what was observed in the study of Mugenzi et al. [19] where sequencing of promoter regions of *CYP6P9a* revealed the existence of haplotypes forming four geographical clusters: southern Africa, East-Central Africa (Kenya/Uganda/Cameroon), West-Central Africa (Benin/DRC), and in Ghana only (West Africa). This further confirms that pyrethroid resistance in *An. funestus* is driven by the selection and overexpression of the genes found in the *rp1* quantitative trait locus (QTL), a locus that has been shown to account for more than 85% of pyrethroid resistance in *An. funestus* [33, 34]. In Ghana, a notable increase in the frequencies of the *6P4a-I*^*220*^*F*^*221*^*M*^*228*^*F*^*406*^*I*^*407*^*I*^*409*^ major allele from 7 to 78% and the *6P4b-E*^*284*^ major allele from 44 to 100*%* for samples collected in the years 2014 and 2021 was observed. This increase coincides with the significant decrease in pyrethroid susceptibility from 36.11 ± 3.87% mortality in 2014 [14] to 11.6 ± 5% in 2021 [15], for the same locality (Atatam, Ghana). The directional selection of these genes under insecticide pressure is similar to the reduced diversity observed in genes found in the *rp1* locus like the tandemly duplicated *CYP6P9a* and *CYP6P9b* [28] but contrasts with the high diversity of genes not found in the *rp1* like the *CYP325A* gene involved in pyrethroid resistance in Cameroon [29] and *CYP6M7*, driving pyrethroid resistance in Zambia [35], both found in the *rp2* QTL, and *CYP9J11* found in the *rp3* QTL [36]. In the southern African population, a remarkable low diversity was observed in 2014 with the existence of major alleles already, which became fixed in 2021. However, the lower expression of *CYP6P4a* and *CYP6P4b* relative to *CYP6P9a and CYP6P9b* [37] suggests that the alleles are not the major drivers of resistance and could have been selected along with the *CYP6P9a* and *CYP6P9b* genes—the major drivers of pyrethroid resistance in this region [18, 33] via genetic hitchhiking. For Eastern (Uganda) and Central Africa (Cameroon), *CYP6P4a* and *CYP6P4b* were highly diversified, with no signs of selection in 2014. In 2021, there is the existence of a common major allele for each gene (*6P4a- R*^*169*^*E*^*295*^*K*^*297*^ and *6P4b-N*^*288*^) which could be as a result of recent gene flow from Eastern to Central Africa [38]. The identification of directional selection and polymorphism in the *CYP6P4a* and *CYP6P4b* genes highlights the adaptive nature of mosquito populations in response to insecticide pressure. Continued monitoring of these genes and their variations will contribute to decipher mosquito evolution, gene flow, and the spread of resistance. This knowledge can inform future strategies for controlling and managing resistance in mosquitoes.

### The directionally selected CYP6P4a and CYP6P4b alleles mediate insecticide resistance via mechanisms involving gene duplication, allelic variation, and gene overexpression

#### The CYP6P4a is duplicated in Ghana

The confirmation of the duplication of *CYP6P4a* by qRT-PCR partially explains the overexpression of the gene and further enlightens on the composition of the previously reported 6.2 kb insertion in Ghana [39]. It could also account for the nearly 2X expression of *CYP6P4a* over *CYP6P4b* (44.8- and 23.9-fold) from published transcriptomic data [18]. Interestingly, the duplication is specific to Western Africa (Ghana), as qPCR evaluation of gene duplication in Southern Africa had revealed no duplication, neither for *CYP6P4a* and *CYP6P4b* nor *CYP6P9a and CYP6P9b* in Malawian and Mozambican *An. funestus* [37]. This 6.2 kb insertion coming with the duplication of *CYP6P4a* could therefore be a response mechanism to insecticide pressure and could contribute to the high pyrethroid resistance now observed in West African *An. funestus* population. Further study on the entire composition of the insertion would help evaluate its impact on the high pyrethroid resistance levels observed in the field. Although overexpression of cytochrome P450 in mosquitoes is more often associated with changes in the *cis*- or *trans*-acting regulatory loci [18, 19, 32, 38], the duplication of *CYP6P4a* here adds to growing evidence that copy number variation is also a major contributor. This has been shown recently in the other major malaria vector *An. gambiae* [40, 41].

### Allelic variation enhances CYP6P4a and CYP6P4b pyrethroid detoxification activity

#### The CYP6P4a mutant allele has greater binding affinity for pyrethroids

In silico modelling and docking has been an essential tool in the characterisation of enzyme–substrate interactions of many P450s [42–44]. The hydroxylation or hydrolysis of the 4′-phenoxybenzyl moiety along with the cis/trans methyl spot of type I (permethrin) [45] and type II (deltamethrin) [28, 46] pyrethroids are reported to be the main routes for pyrethroid metabolism. In the implemented docking protocol of this study, the 4′ hydroxylation of the phenoxybenzyl moiety, which is the major pyrethroid metabolism route, was indeed more enriched compared to other minor metabolic routes aligning with previously reported structural experiments [46]. Molecular docking and thermodynamic integration simulation showed that the major *CYP6P4a* allele in Ghana (mutant) exhibits higher affinity for permethrin and deltamethrin than the susceptible lab strain FANG allele (wildtype), underscoring the key role of non-synonymous mutations in insecticide resistance. Mutations in the 6P4a-GHA model were predicted to induce structural backbone deviations in the active site architecture within SRS-1 and 6 regions, resulting in stronger binding affinity in the 6P4a-GHA model. SRS motifs in P450 enzymes play a crucial role in the metabolism of insecticides. Deltamethrin preference for hotspot residues SRS-1 (*S*^*119*^,* H*^*121*^) and SRS-6 (*F*^*495*^) as well as permethrin bonding with SRS-1 (*R*^*107*^, *H*^*121*^) and SRS-6 (*F*^*495*^) on the mutant (6P4a-GHA) allele were the main discriminative patterns in pyrethroids binding modes to the wild-type vs. mutant-type models and may suggest a causal link with the 6P4a-GHA mutations. In agreement with this, previous studies [47] investigated the significance of conserved SRS-1 CYP3A4 residues through site-directed mutagenesis. The findings highlighted the critical role of the highly conserved residue *S*^*119*^ in determining specificity in the active site topology in steroid 6beta-hydroxylation. Interestingly, both pyrethroids were predicted to bind more mutant SRS-1 residues than the wild-type. Also, the SRS-6 region of CYP6AE11-20 has been studied using in vitro metabolism and modelling techniques. It was found that amino acid substitutions at position 495 in SRS-6 led to significant alterations in the shape and chemical environment of P450 enzyme active site [48]. Interestingly, our findings reveal that the structural changes induced by the 6P4a-GHA model mutations in the SRS-6 region, particularly at residue *F*^*495*^, result in a favourable conformation that enhances substrate selectivity for permethrin over the wild-type case. Similar substrate affinity was observed with the *An. arabiensis CYP6P4* orthologue [49] but contrary to what was reported for *CYP6P12*, the *CYP6P4* orthologue in *Ae. albopictus* [50], where the 4′ hydroxylation of the phenoxybenzyl moiety was not a detoxification route. While most of these mutations mapped to locations within or adjacent to the active site, the CYP6P4b-284E mapped to an external loop at considerable distance away from the catalytic site, making it difficult to predict the functional impact of the mutation through molecular docking alone. However, mutations in surface residues have been shown to influence electron transfer rates from P450 reductase and/or cytochrome b5 [51, 52], and this could be a potential mechanism involved with this mutation.

#### In vitro*, *CYP6P4a and CYP6P4b mutant alleles were better metabolisers of pyrethroids

The patterns of differential enzyme–substrate interactions revealed via molecular docking were confirmed with in vitro insecticide metabolism assay. Recombinant enzymes exhibited direct metabolism of types I and II pyrethroid, with more metabolism observed for deltamethrin as predicted by the molecular docking experiment. Metabolism chromatograms displayed distinct metabolite peaks, which we predict to correspond to deltamethric acid, cyano(3-hydroxyphenyl)methyl deltamethrate, and 4′-hydroxy-deltamethrin (Fig. 3A). These findings support the fact that the P450 enzymes metabolise pyrethroids preferentially via ring hydroxylation [46]. Nevertheless, the precise identification of the metabolites from *CYP6P4a/b* insecticide depletion requires further experimental procedures like mass spectroscopy and nuclear magnetic resonance. The Ghana *6P4a-I*^*220*^*F*^*221*^*M*^*228*^*F*^*406*^*I*^*407*^*I*^*409*^ and *6P4-E*^*284*^ alleles exhibited significantly higher depletion compared to the FANG variants *6P4a-N*^*286*^*R*^*289*^*S*^*291*^ and *6P4b-T*^*291*^*V*^*294*^*Y*^*399*^ suggesting that the selection and overexpression of these alleles in the field mosquitoes could be as a result of their increased metabolic detoxification activity against insecticides. Orthologues of these genes have shown similar profiles. For example, *An. gambiae s.s. CYP6P4* metabolise type I and type II pyrethroids [40], whereas *An. arabiensis CYP6P4* metabolise permethrin [49]. The *CYP6P4a* Malawi allele also exhibited high depletion activity; its contribution to resistance might be limited due to its lower expression level compared to *CYP6P9a*/b genes—the major drivers of pyrethroid resistance in southern Africa. Allelic variation as a mechanism in insecticide resistance has also been reported in *CYP6a2*, in *D. melanogaster* [53], *GSTe2* in association with DDT resistance in *An. funestus* [27] and *Ae. aegypti* [54], and *CYP6P9a*/*b*, in association with pyrethroid resistance in *An. funestus* [28]*.* For *CYP6P4a*, the *I*^*220*^*F*^*221*^*M*^*228*^*F*^*406*^*I*^*407*^*I*^*409*^ haplotype found in Ghana accounted for the increased detoxification activity observed. Site directed mutagenesis experiments could help identify the mutation(s) accounting for the greatest difference in enzyme activity. For *CYP6P4b*, the *284E* mutation impacts enzyme activity and the simple reversal of the amino acid to D284 could help better quantify the biochemical impact of the mutation. Such experimental procedures were used to evaluate the impact of key mutations in *An. funestus CYP6P9a* and *CYP6P9b* [28, 55] and in human *CYP2D6* [56].

#### In vivo*, *allelic variation and overexpression of CYP6P4a and CYP6P4b impact pyrethroid insecticide resistance

In West Africa, where *CYP6P4a* and *CYP6P4b* are overexpressed, the consequential impact of the overexpression of the mutant alleles in the field was validated, with higher resistance to pyrethroids recorded in the transgenic flies compared to the non-transgenics. The phenotypic impact of mutations under selection in this population was revealed as flies overexpressing the mutant alleles (Ghana) showed lower mortality compared to those overexpressing the wild-type (FANG). This shows that the mutant Ghana allele has a selective advantage in the field over the wildtype allele in the presence of high pyrethroid use due to its higher metabolic efficiency. These mechanisms are similar to those reported for the *CYP6P9a* and *CYP6P9b* sister genes [28] and also for *GSTe2* conferring DDT resistance [27], but with the *CYP6P4* orthologue in *Ae. albopictus (CYP6P12)*, it was demonstrated that overexpression of the gene conferred resistance to deltamethrin but increased mortality to permethrin [50]. This study therefore validates that *CYP6P4a* and *CYP6P4b* together exacerbate pyrethroid resistance in Ghana via mechanisms of allelic variation and overexpression.

### The CYP6P4b-D284E and the CYP6P4a-M220I together exacerbate resistance to pyrethroids

The CYP6P4a-M220I and CYP6P4b-D284E molecular markers were strongly associated with pyrethroid resistance and a significant decrease in the killing effect of pyrethroid-only nets (Olyset, PermaNet 2.0, and DuraNet), where homozygous mutants and heterozygotes for the two alleles survived more compared to wild-types. This is similar to the impact of *CYP6P9a* and *CYP6P9b* markers on the efficacy of pyrethroid bed nets [18, 19, 32]. It is now imperative to assess the influence of these markers on the effectiveness of nets containing the synergist piperonyl butoxide (PBO) and on dual-action nets that combine pyrethroids with other insecticides in semi-field conditions, such as experimental huts to further evaluate the effectiveness of various control tools. Also, it is important to investigate the roles of these alleles in cross-resistance to other insecticide classes including organochlorines, organophosphates, carbamates, neonicotinoids, and insect growth regulator. In effect, studies on resistance to DDT in West Africa showed that *CYP6P4a* and *CYP6P4b* were the most overexpressed detoxification genes in DDT-resistant mosquitoes [57] as well as in carbamate-resistant mosquitoes [58], but their implication in the phenotype needs to be validated. These evaluations will guide the selection of optimal insecticide-based vector control tools in regions where resistance is driven by *CYP6P4a* and *CYP6P4b*.

#### The CYP6P4b-D284E and the CYP6P4a-M220I alleles are prominent in West Africa

The new DNA-based markers designed here can be effectively integrated into resistance management strategies through routine monitoring and tracking of the spread of *CYP6P4a/b*-driven insecticide resistance in the field. Analysis of the geographical distribution of these markers has revealed their high prevalence in sampled populations from Ghana, Guinea, Sierra Leone, and Benin, with their prevalence steadily increasing over time. These mutations were notably absent in other regions of Africa, highlighting their association with West Africa and the restriction of gene flow among populations of this species between the major geographical regions [30, 39]. Nonetheless, the absence of the *CYP6P4a* and *CYP6P4b* markers described herein in a population does not directly correlate with absence of insecticide resistance. This is because as evidenced in this study, distinct alleles of *CYP6P4a* and *CYP6P4b*, differing from those observed in West Africa, have been selected in other regions and are likely conferring resistance but which the markers developed in this study cannot detect. Additionally, alternative resistance mechanisms such as target site resistance, cuticular resistance [59], and other metabolic resistance genes could be driving resistance in other localities. For example, the *CYP6P9a* and *CYP6P9b* resistance alleles drive insecticide resistance in Southern Africa [18, 19, 32], and the *CYP9K1* resistance allele (454A-CYP9K1) drives resistance in East-Central Africa [17]. It is however imperative to continue assessing the distribution of the CYP6P4a-M220I and CYP6P4b-D284E markers in other West African countries where pyrethroid resistance is prevalent, while also maintaining continuous monitoring in other African regions to detect potential gene flow or de novo occurrence. This proactive strategy will guide effective resistance management strategies before the alleles reach high frequencies, thereby contributing to maintaining the efficacy of pyrethroid-based control tools and, ultimately, to malaria elimination efforts.

## Conclusions

This study made use of a combination of computational analysis and laboratory experiments to provide in-depth insights into the molecular mechanisms by which the duplicated *CYP6P4a* and *CYP6P4b*, the most overexpressed detoxification genes in pyrethroid-resistant *An. funestus* in West Africa, drive pyrethroid metabolic resistance. The study provided evidence that *CYP6P4a* and *CYP6P4b* confer insecticide resistance in the malaria vector *An. funestus* via mechanisms involving gene overexpression and allelic variation. The identification of molecular markers of insecticide resistance within these genes that compromise the effectiveness of pyrethroid-based vector control tools represents a significant input in the resistance management toolbox of malaria vector control bodies in West Africa. We encourage the deployment of nets incorporating piperonyl butoxide (PBO) or chlorfenapyr for enhanced impact, while also encouraging the development of alternative mosquito management strategies. Additionally, we emphasise the importance of conducting further investigations into the underlying mechanisms and genes associated with resistance. This knowledge will be pivotal in the development of improved insecticides that will enhance vector control measures.

## Methods

### Mosquito samples

For this study, mosquitoes were collected between 2018 and 2021 from the following localities: Mibellon in Cameroon (6° 4′ 60″ N, 11° 70′ 0″ E) [60], Mayuge in Uganda (0° 23′ 10.8″ N, 33° 37′ 16.5″ E) [61], Obuasi in Ghana (06° 17.377″ N, 001° 27.545″ W) [15], Chikwawa in Malawi (16° 1′ S; 34° 47′ E), and Palmeira in Mozambique (25° 15′ 19″ S; 32° 52′ 22′″ E) [19]. All *F*_0_ were identified as *An. funestus* using *Anopheles funestus* cocktail PCR [62], and the resistance profiles of the populations were as reported in the cited articles. Two *An. funestus* laboratory colonies were also used in this study: the FANG colony, which is a fully insecticide-susceptible colony originating from Angola and maintained in laboratory conditions, and the FUMOZ colony, which is a multi-insecticide-resistant colony originating from southern Mozambique, all maintained under laboratory conditions since early 2000 [63]. The above mosquitoes have been subjected to insecticide bioassays, with their resistance profiles published in the above studies. The F1 female mosquitoes alive 24 h post-exposure were stored in – 80 °C and were used for this study.

### Africa-wide polymorphism analysis of An. funestus CYP6P4a and CYP6P4b

To investigate the patterns of genetic variability and potential signatures of selection of *CYP6P4a* and *CYP6P4b* in *An. funestus* across Africa, the full-lengths of *CYP6P4a* and *CYP6P4b* were amplified from pyrethroid-resistant female mosquitoes from the aforementioned countries and from FANG and FUMOZ to be used as references for susceptible and resistant alleles. Amplicons were purified, cloned, and sequenced as described in Additional File 3: Method 1. Nucleotide polymorphisms were identified through manual examination of sequences and multiple sequence alignments using BioEdit 7.0.5 [64]. Population genetics parameters of polymorphism like nucleotide diversity π, haplotype diversity, and Tajima’s *D* and Fu and Li Tajima D* selection estimates of both genes were determined using the DnaSP 5.1 software [65]. Different haplotypes of the genes were compared by constructing a maximum likelihood phylogenetic tree using MEGAX [66]. The best-fit substitution model based on the Bayesian information criterion that best described the haplotype dataset was the Jones-Taylor-Thornton Gamma distribution model. This was then used to generate the maximum likelihood tree with 1000 bootstrap replications to assess the robustness of the tree. Additionally, haplotype networks were constructed using the TCS programme [67] to assess the connection between the various haplotypes and pyrethroid resistance.

#### Assessment of the temporal variation of the CYP6P4a and CYP6P4b resistance alleles in Africa

In order to evaluate the temporal dynamics of *CYP6P4a* and *CYP6P4b*, we performed a temporal comparison of the diversity of the genes between 2014 and 2021. Polymorphism pattern of the genes was analysed across Africa using a previously generated SureSelect dataset, which included samples collected in 2014 from Uganda, Malawi, and Cameroon [68]. The *CYP6P4a and CYP6P4b* polymorphisms had been extracted from the SNP Multisample report file generated through Strand NGS 3.4 for each population. BioEdit [64] was used to introduce different polymorphisms into the VectorBase reference sequence, while ambiguity codes were employed to indicate heterozygote positions in the sequences. Samples analysed include Malawi, Uganda, Cameroon, FANG, and FUMOZ. For the Ghana population, the genes from permethrin resistant mosquitoes from a 2014 collection were cloned and sequenced. Haplotype reconstruction and polymorphism analyses were conducted using DnaSP 5.1 [65], and MEGAX [66] was used to construct the maximum likelihood phylogenetic trees for both *CYP6P4a* and *CYP6P4b* genes.

### Assessment of copy number variation of CYP6P4a in Ghana

Genome-wide analyses of the *An. funestus* population of Ghana revealed the presence of a 6.2 kb insertion between *CYP6P4a* and *CYP6P5* [39]. Quantitative PCR (qPCR) was used to assess whether a potential copy-number variation or gene amplification of *CYP6P4a* could be associated with its significant upregulation in resistant mosquitoes. Genomic DNA (gDNA) from Ghana mosquitoes and FANG were extracted following the Livak method [69]. The gDNA concentration and integrity were assessed using an Implen nanophotometer N50 (Implen, Munich, Germany). The concentrations were adjusted to 14 ng/μL, and 1 μL of gDNA was used in a qPCR reaction for the quantification of *CYP6P4a* (Primers in Table S8). The fold change of the gene in Ghana compared to FANG was calculated using the 2^−∆∆CT^ equation [70] after normalisation with housekeeping genes Actin (AFUN006819-RA) and RSP7 (ribosomal protein S7; AFUN007153-RA).

### In silico characterisation of CYP6P4a and CYP6P4b

To predict the impact of detected polymorphisms on the enzyme structure and enzyme-insecticide interactions, we performed computational modelling of CYP6P4a and molecular docking of pyrethroid ligands within the active sites of modelled enzymes.

#### Homology modelling of protein structures

The 3D atomic resolution structures of the major resistant alleles identified in Ghana (CYP6P4a*-*GHA) and one from FANG (CYP6P4a-FANG) were modelled using AlphaFold [71], with the default settings implemented in Python 3 running on T4 GPU system. Of three retained models predicted for each allele, models with the highest average estimated reliability score (pLDDT: predicted local distance difference test) were selected for downstream simulations. In addition, model quality was assessed against the crystal structure of human microsomal P450, *CYP3A4*(PDB 1TQN) as a template [72], with 33.82% and 33.40% identity to CYP6P4a-GHA and CYP6P4a-FANG query sequences respectively. The ligand structures of deltamethrin (PubChem CID: 40,585) and permethrin (PubChem CID: 40,326) were retrieved from the PubChem database (https://pubchem.ncbi.nlm.nih.gov/). Structure preparation and suitability for docking is as described in Additional File 3: Method 2 [73].

#### Molecular docking of CYP6P4a and CYP6P4b with pyrethroids

The Dock module implemented in MOE was used for docking computations. First, the docking protocol was optimised and validated by varying pairs of scoring function parameters. This was achieved by re-docking the native heme molecule with its apo CYP3A4 structure and to heme-mapped binding pockets of predicted models, aiming at reproducing the original heme binding pose. The scoring function pair with optimal performance was retained and include Affinity ∆G for preinitial-scoring and the generalized Born volume integral/weighted surface area (GBVI/WSA) for rescoring [74]. GBVI/WSA is an AMBER99 forcefield [75] parameterised function trained on 99 experimentally determined protein–ligand complexes and known to estimate binding free energies of complexes with great accuracy. The retained scoring functions consistently re-produced heme binding poses with negligible deviations (RMSD < 0.5Å) between predicted and experimental binding modes. Secondly, optimised parameters were applied to dock insecticides separately with the respective targets. Substrate recognition site (SRS) residues above the heme catalytic centre were used to define the binding pockets of the models and include the following: SRS1 (H121, F123, A124), SRS2 (F213), SRS4 (F310, V311, L314), SRS5 (I381), SRS6 (S494, F495). In addition, the Ghana mutations L406F, V407I, and L409I were also included in the binding site due to proximity. A 250-ns thermodynamic integration simulation was subsequently performed to validate molecular docking results. Structural analyses and visualisation were performed with the PyMOL and MOE.

### Heterologous expression of alleles in E. coli and insecticide metabolism assay

We investigated the enzymatic activity of recombinant CYP6P4a and CYP6P4b enzymes in metabolising pyrethroid insecticides to validate their direct involvement in the phenotype and the impact of allelic variation on enzyme activity.

#### Cloning of CYP6P4a and CYP6P4b candidate alleles

Candidate *CYP6P4a* and *CYP6P4b* alleles were cloned and expressed in *E. coli* cells and used for insecticide metabolism assays. The expression constructs were designed based on previous work [76]. Briefly, expression plasmids were constructed (primers in Table S8) by fusing cDNA fragment from a bacterial ompA + 2 leader sequence with its downstream ala-pro linker to the NH_2_-terminus of the P450 cDNA, in frame with the P450 initiation codon, as described in previous studies [28, 37, 76]. These constructs were digested with the *NgoM*IV and *Xba*I restriction enzymes and ligated to pCW-ori + expression vector, linearised with the same restriction enzymes, creating the constructs pB13::OMPA + 2-CYP6P4a for CYP6P4a sequences (CYP6P4a-GHA, CYP6P4a-FANG, and CYP6P4a-MWI, for Ghana, FANG, and Malawi alleles, respectively). Similarly, the constructs pB13::OMPA + 2-CYP6P4b were created for *CYP6P4b* sequences (CYP6P4b-GHA, CYP6P4b-FANG, CYP6P4b-MOZ, and CYP6P4b-UGA, for Ghana, FANG, Mozambique, and Uganda alleles, respectively).

#### Heterologous expression of recombinant CYP6P4a and CYP6P4b

The *E. coli JM109* cells were co-transformed with the above P450 constructs and a plasmid containing the *An. gambiae* cytochrome P450 reductase (pACYC-AgCPR). Co-expression was induced following standard protocols and as described in Additional File 3: Method 3 [28, 46, 77].

#### Insecticide metabolism assays and HPLC analysis

Insecticide metabolism assay was carried out following previously described method [28]. Briefly, recombinant enzymes were incubated with pyrethroid insecticides in the presence of a NADPH source. Reverse-phase high-performance liquid chromatography (HPLC) was used to monitor the depletion of the substrate and the emergence of metabolites. Detailed procedure of the metabolism assay and is found in Additional File 3: Method 4.

### In vivo transgenic expression of CYP6P4a and CYP6P4b in Drosophila melanogaster flies

To investigate if overexpression of *CYP6P4a* and *CYP6P4b* alone can confer pyrethroid resistance to a model organism and establish if the intensity of the resistance is impacted by allelic variation, *CYP6P4a* and *CYP6P4b* were expressed in *Drosophila* flies using the GAL4/UAS system [37, 78]. Transgenic lines for *CYP6P4a* (UAS-CYP6P4a-GHA and UAS-CYP6P4a-FANG) and *CYP6P4b* (UAS-CYP6P4b-GHA, UAS-CYP6P4b-FANG, and UAS-CYP6P4b-MOZ) were generated and subjected to pyrethroid susceptibility tests. The procedure for the generation of constructs to make up the experimental group and the control group (non-transgenic) is described in Additional File 3: Method 5 [28, 37].

#### Insecticide susceptibility contact assays

Five replicates of F_1_ female flies (2–5 days old) from experimental and control groups were exposed to pyrethroids (4% permethrin-, 0.2% deltamethrin-, and 0.0007% alphacypermethrin-impregnated filter papers prepared in acetone and Dow Corning 556 Silicone Fluid (BHD/Merck, Germany) for a period of 24 h, with mortality plus knockdown recorded after 1, 2, 3, 6, 12, and 24 h. A comparison of the mortality rates between the experimental groups and the control groups was used to assess if candidate genes’ overexpression alone is enough to confer resistance, while a comparison with flies harbouring the FANG allele shed light on the impact of allelic variation on resistance.

To confirm the overexpression of candidate genes in the experimental group as opposed to the control group, qRT-PCR was carried out using RNA extracted from three replicates of pools of five experimental and control female flies. Details of the qRT-PCR reaction and gene expression quantification is found in Additional File 3: Method 6.

### Design of DNA diagnostic marker assays for CYP6P4a and CYP6P4b mutations

A single, most predominant mutation identified in the Ghana variants of *CYP6P4b* (D284E) and *CYP6P4a* (M220I) was targeted to develop DNA-based diagnostic assays (CYP6P4b-D284E and CYP6P4a-M220I). For *CYP6P4b*, the design exploited the Amplification Refractory Mutation System (ARMS) PCR principle (http://primer1.soton.ac.uk/primer1.html) that allowed the specific amplification of the wild-type allele (FANG) and the mutant allele (Ghana). For *CYP6P4a*, a locked nucleic acid (LNA) probe-based PCR (Integrated DNA technologies, UK) was designed to discriminate between the wild-type and mutant alleles of *CYP6P4a.* Details of PCR reactions for both assays are described in Additional File 3: Method 7.

### Association between the CYP6P4a and CYP6P4b markers and pyrethroid resistance

The high level of pyrethroid resistance, coupled with the high frequency of the resistance alleles in the Ghanaian field population, made the establishment of the link between the genotype and the resistance phenotype difficult. To address this challenge, a mosquito genetic cross between female FANG bearing the homozygote susceptible (SS) CYP6P4b D284/D284 and CYP6P4a M220/M220 genotypes and male field mosquitoes from Ghana bearing the homozygote resistant (RR) CYP6P4b E284/E284 and CYP6P4a I220/I220 genotypes was established as done for the development of *CYP6P9a*/b markers [18, 19]. The crosses were maintained through to the fourth generation to allow the three genotypes (RR, RS, and SS) to segregate. WHO tube bioassays were carried out on 3- to 5-day-old hybrid female mosquitoes using pyrethroid insecticide papers following WHO protocol [5], and the correlation between resistance phenotype and genotypes was established using odds ratio.

### Impact of CYP6P4a and CYP6P4b resistance alleles on the bio-efficacy of bed nets

The effectiveness of current pyrethroid-treated bed nets in relation to the impact of the *CYP6P4a* and *CYP6P4b* resistant alleles was assessed using cone bioassays [79]. Bed nets evaluated were Olyset Net (1000 mg/m^2^ permethrin), PermaNet 2.0 (55 mg/m^2^ deltamethrin), and DuraNet (261 mg/m^2^ alpha-cypermethrin). Five replicates of ten females (2- to 5-day old) from F_3_ and F_4_ generations were placed in plastic cones enclosed with the aforementioned bed nets for 3 min. Post-exposure to the bed nets, mosquitoes were transferred to holding paper cups and fed with cotton soaked in a 10% sugar solution. Final mortality rates were assessed 24 h later, and DNA was extracted from 30 to 40 alive and dead mosquitoes using the LIVAK protocol, and the genotypes were determined. Mortality and the correlation between resistance phenotype and *CYP6P4a* and *CYP6P4b* genotypes were used to evaluate the efficacy of the bed nets.

### Africa-wide distribution of CYP6P4a and CYP6P4b resistance alleles

The Africa-wide geographical distribution of the *CYP6P4a* and *CYP6P4b* resistance alleles was assessed by genotyping genomic DNA samples of female *An. funestus* collected in different regions of Africa in different years—Central Africa: Cameroon-Mibellon (2021); Southern Africa: Malawi-Chikwawa (2021); Eastern Africa: Uganda-Mayuge (2022), Tanzania-Maheza (2017), 38° 54′ 14.9904 ″ E; https://www.countrycoordinate.com/; and Western Africa: Benin (2022), Seirra-Leone-Largo (2021), 12° 8′ 56.32″ W, https://www.longitude-latitude-maps.com/city/, and Guines-Yome (2023). Twenty-three to forty parental mosquitoes were genotyped per locality using the above diagnostic tools.

### Statistical analysis

Statistical analysis was done using GraphPad Prism 7. Statistical significance was set at 0.05 and 95% confidence interval (CI). Comparison of the insecticide depletion of enzymes and transgenic insecticide contact bioassay was done using Student *t*-test. The correlation between the CYP6P4a_R and the CYP6P4b_R markers and their impact on pyrethroid resistance phenotype and on the bio-efficacy of bed nets was established using odds ratio (OR) and Fisher’s exact test.

## Supplementary Information

Additional file 1: Fig. S1. Phylogenetic trees showing high genetic diversity of *CYP6P4a* and *CYP6P4b* in *An. funestus* collected in 2014 in CMR, UGA, MWI, and in FUMOZ and FANG lab strains. Fig. S2 – Haplotype networks showing the genetic diversity patterns of *CYP6P4a* and *CYP6P4b* in *An. funestus* collected in 2014 in CMR, UGA, MWI, and in FUMOZ and FANG lab strains. Fig. S3 – Amino-acid alignment of *CYP6P4a* and *CYP6P4b* variants from *An. funestus* across Africa. Fig. S4 – Modelling of CYP6P4a Ghana and FANG variants and assessment of model quality. Fig. S5 – A two-dimensional representation showing typical pyrethroid orientations with the 4'-phenoxy group approaching the heme iron. Fig. S6 – Graph showing genomic duplication of*CYP6P4a* in *An. funestus *from Ghana. Fig. S7 – Graphs showing expression of candidate P450s, evident by absorption peak occurring at 450nm, and metabolism of insecticides by recombinant CYP6P4a and CYP6P4b enzymes. Fig. S8 – Graph confirming expression of *CYP6P4a* and *CYP6P4b* transgenes in* D. melanogaster. *Fig. S9 – Schematic alignment of sequences across Africa showing SNPs occurring in *CYP6P4a* and *CYP6P4b* in Ghana and graphs showing their distribution in field and laboratory hybrid strain, using newly designed CYP6P4a-M220I and CYP6P4b-D284E molecular diagnostic tools. Fig. S10 – Susceptibility profile of FANG/GHANA strain used in the evaluation of the impact of CYP6P4a-M220I and CYP6P4b-D284E markers on resistance and on bio-efficacy of ITN.

Additional file 2: Table S1. Nucleotide diversity parameters of the coding regions of *CYP6P4a* and *CYP6P4b *in *An. funestus* collected in 2021. Table S2 –Nucleotide diversity parameters of the coding regions of *CYP6P4a* and *CYP6P4b *in *An. funestus* collected in 2014. Table S3 – Characteristics of productive poses binding with pyrethroids 4'-phenoxy spot approaching above the heme iron. Table S4 – Protocol for using CYP6P4b-D284E molecular diagnostic tool. Table S5 – Protocol for using CYP6P4a-M220I molecular diagnostic tool. Table S6 – Association between insecticide susceptibility as determined by WHO tube bioassay and CYP6P4a-M220I and CYP6P4b-D284E genotypes in An. funestus crossing between field and FANG lab colony. Table S7 – Association between insecticide susceptibility as determined by WHO cone bioassay and CYP6P4a-M220I and CYP6P4b-D284E genotypes in *An. funestus* crossing between field and FANG lab colony. Table S8 – List of primers used in the study.

Additional file 3: Method 1. Description of the amplification and cloning of full-length cDNA of *An. funestus CYP6P4a* and *CYP6P4b. *Method 2. Model structure preparation and suitability for docking. Method 3. Heterologous expression of recombinant *CYP6P4a* and *CYP6P4b. *Method 4. Description of insecticide metabolism assays and HPLC analysis. Method 5. Construction and i*n vivo* transgenic expression of *CYP6P4a* and *CYP6P4b* in *Drosophila melanogaster *flies. Method 6. qPCR evaluation of transgenic expression on *D. melanogaster *flies. Method 7. Design of DNA diagnostic marker assays for *CYP6P4a* and *CYP6P4b *mutations.

## Data Availability

cDNA sequences of *CYP6P4a* and *CYP6P4b* generated in this study have been deposited in GenBank [accession numbers: PP667999—PP668085 for *CYP6P4a* and PP694837—PP694908 for *CYP6P4b*]. All data generated or analysed during this study are included in this published article and its additional files.

## Abbreviations

Ae.: Aedes
AL: Alive
An.: Anopheles
bp: Base pair
BEN: Benin
C: Control
cDNA: Complementary DNA
CMR: Cameroon
CYP450s: Cytochrome P450s
DDT: Dichlorodiphenyltrichloroethane
DE: Dead
DNA: Deoxyribonucleic acid
E. coli: Escherichia coli
F1: First filial generation
GHA: Ghana
GST: Glutathione S-transferase
gDNA: Genomic DNA
Hd: Haplotype diversity
HR: Highly resistant
HS: Highly susceptible
HPLC: High-performance liquid chromatography
LNA: Locked nucleic acid
MWI: Malawi
MOZ: Mozambique
PCR: Polymerase chain reaction
qRT-PCR: Quantitative reverse transcriptase PCR
QTL: Quantitative trait locus
RR: Homozygous mutant or resistant
RMSD: Root mean square deviation
RS: Heterozygous mutant
SS: Homozygous wildtype or susceptible
SNP: Single-nucleotide polymorphism
SRS: Substrate recognition site
RNA: Ribonucleic acid
RPL11: Ribosomal protein L11
RSP: Ribosomal protein
s.l.: Sensu lato
UGA: Uganda
