# Supplementary material for: Two highly selected mutations in the tandemly duplicated CYP6P4a and CYP6P4b genes drive pyrethroid resistance in Anopheles funestus in West Africa
Source: BMC Biol. 2024 Dec 18;22:286. doi: 10.1186/s12915-024-02081-y (PMC11657943; doi:10.1186/s12915-024-02081-y)
Supplement: Supplementary file 1 — Additional file 1: Fig. S1. Phylogenetic trees showing high genetic diversity of CYP6P4a and CYP6P4b in An. funestus collected in 2014 in CMR, UGA, MWI, and in FUMOZ and FANG lab strains. Fig. S2 – Haplotype networks showing the genetic diversity patterns of CYP6P4a and CYP6P4b in An. funestus collected in 2014 in CMR, UGA, MWI, and in FUMOZ and FANG lab strains. Fig. S3 – Amino-acid alignment of CYP6P4a and CYP6P4b variants from An. funestus across Africa. Fig. S4 – Modelling of CYP6P4a Ghana and FANG variants and assessment of model quality. Fig. S5 – A two-dimensional representation showing typical pyrethroid orientations with the 4'-phenoxy group approaching the heme iron. Fig. S6 – Graph showing genomic duplication ofCYP6P4a in An. funestus from Ghana. Fig. S7 – Graphs showing expression of candidate P450s, evident by absorption peak occurring at 450nm, and metabolism of insecticides by recombinant CYP6P4a and CYP6P4b enzymes. Fig. S8 – Graph confirming expression of CYP6P4a and CYP6P4b transgenes in D. melanogaster. Fig. S9 – Schematic alignment of sequences across Africa showing SNPs occurring in CYP6P4a and CYP6P4b in Ghana and graphs showing their distribution in field and laboratory hybrid strain, using newly designed CYP6P4a-M220I and CYP6P4b-D284E molecular diagnostic tools. Fig. S10 – Susceptibility profile of FANG/GHANA strain used in the evaluation of the impact of CYP6P4a-M220I and CYP6P4b-D284E markers on resistance and on bio-efficacy of ITN. [file 12915_2024_2081_MOESM1_ESM.docx]

**Supplemental Information for:**

**Two highly selected mutations in the tandemly duplicated *CYP6P4a* and *CYP6P4b* drive pyrethroid resistance in *Anopheles funestus***

Nelly M.T. Tatchou-Nebangwa^1,2*^, Leon M. J. Mugenzi^1,5^, Abdullahi Muhammad^3,7^, Derrick N. Nebangwa^4^, Mersimine F.M. Kouamo^1^, Carlos S. D. Tagne^1,6^, Theofelix A. Tekoh^1,2^, Magellan Tchouakui^1^, Stephen M. Ghogomu^2^, Sulaiman S. Ibrahim^1,8^, and Charles S. Wondji^1,3*^

^1^ Centre for Research in Infectious Diseases (CRID), P.O. BOX 13591, Yaounde, Cameroon.

^2^ Department of Biochemistry and Molecular Biology, Faculty of Science, University of Buea, P.O. Box 63, Buea, Cameroon.

^3^ Vector Biology Department, Liverpool School of Tropical Medicine (LSTM), Pembroke Place, Liverpool, L3 5QA, UK.

^4^ Randall Centre for Cell and Molecular Biophysics, Faculty of Life Sciences and Medicine, King’s College London, UK.

^5^ Syngenta Crop Protection, Werk Stein, Schaffhauserstrasse, Stein CH4332, Switzerland 6Lead.

^6^ Department of Biochemistry, Faculty of Science, University of Bamenda, Bamenda, Cameroon.

^7^ Centre for Biotechnology Research, Bayero University, Kano, PMB, 3011, Kano Nigeria.

^8^ Department of Biochemistry, Bayero University, PMB, 3011, Kano, Nigeria.


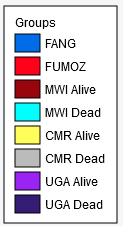


**A**

**B**

**Fig. S1. Genetic diversity of patterns of the coding region of *CYP6P4a* and *CYP6P4b* in 2014.** **A.** Phylogenetic tree for *CYP6P4a* from 2014 sureselect data across Cameroon, Uganda, Malawi*;* **B**. Phylogenetic tree for *CYP6P4b* from 2014 sureselect data across Cameroon, Uganda, Malawi


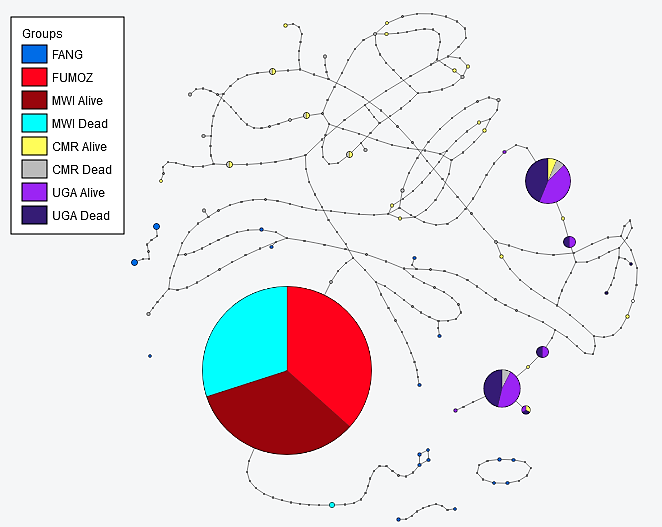

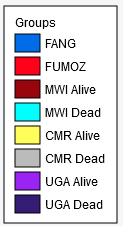

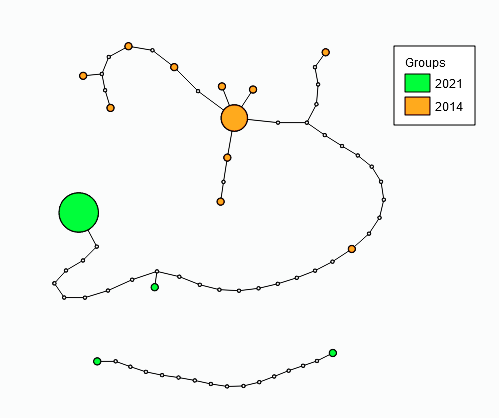

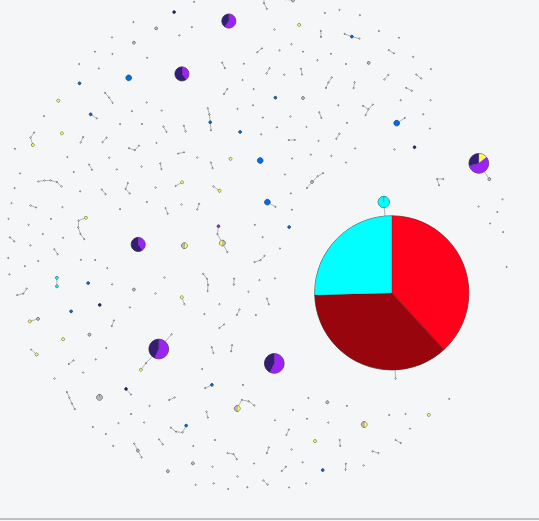


**A**

**D**

**B**

**C**


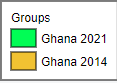

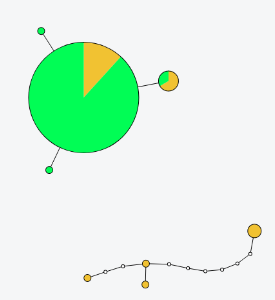


**Fig. S2 Genetic diversity of patterns of the coding region of *CYP6P4a* and *CYP6P4b* in 2014.** **A**. TCS haplotype network for *CYP6P4a* from 2014 sureselect data across Cameroon, Uganda, Malawi. A fixed haplotype is observed in Southern population (Malawi and FUMOZ) and the formation of major haplotypes in Uganda, but high diversity in Cameroon; **B.** TCS haplotype network for *CYP6P4a* from Ghana 2014 and 2021 samples showing high diversity in 2014 but selection of major resistance haplotypes in 2021; **C.** Haplotype network for *CYP6P4b* from 2014 sureselect data across Cameroon, Uganda, Malawi. Similarly, A fixed haplotype is observed in Southern population and the formation of major haplotypes in Uganda, but very high diversity in Cameroon; **D**. Haplotype network for *CYP6P4b* from Ghana 2014 and 2021 samples showing high diversity in 2014 but with the presence of resistance alleles that have undergone selection to near fixation in 2021.


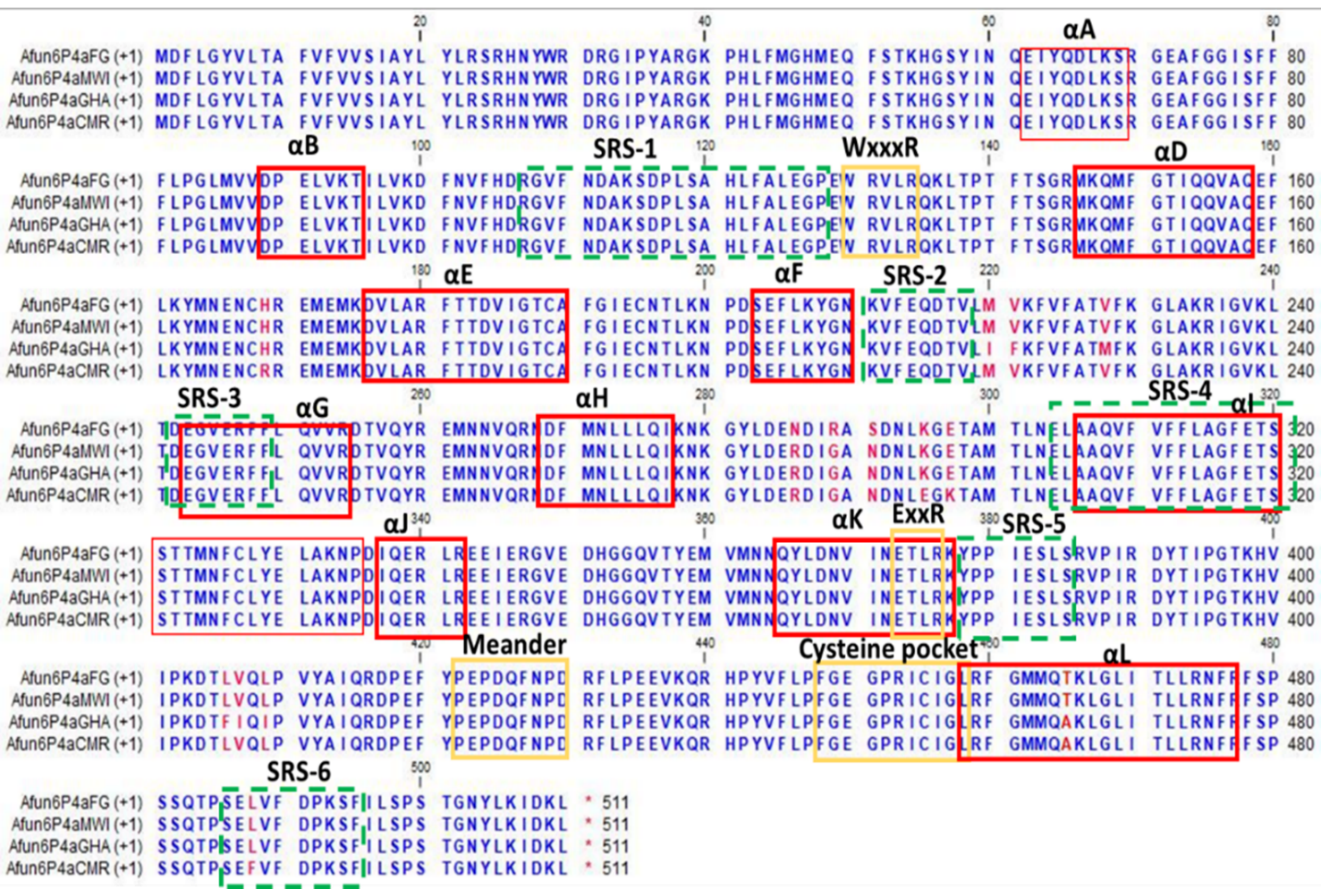


**Fig. S3A.** **A. Amino-acid alignment of *CYP6P4a* variants highlighting substitutions.** Key conserved P450 motifs and substrate recognition sites are annotated. The solid red lines represent helices A-L, while dashed blue lines correspond with the substrate recognition sites 1-6. Solid orange lines identify the structurally conserved motifs of the CYP450s. Variable residues are in hot pink. Residues 310-315 corresponds to the


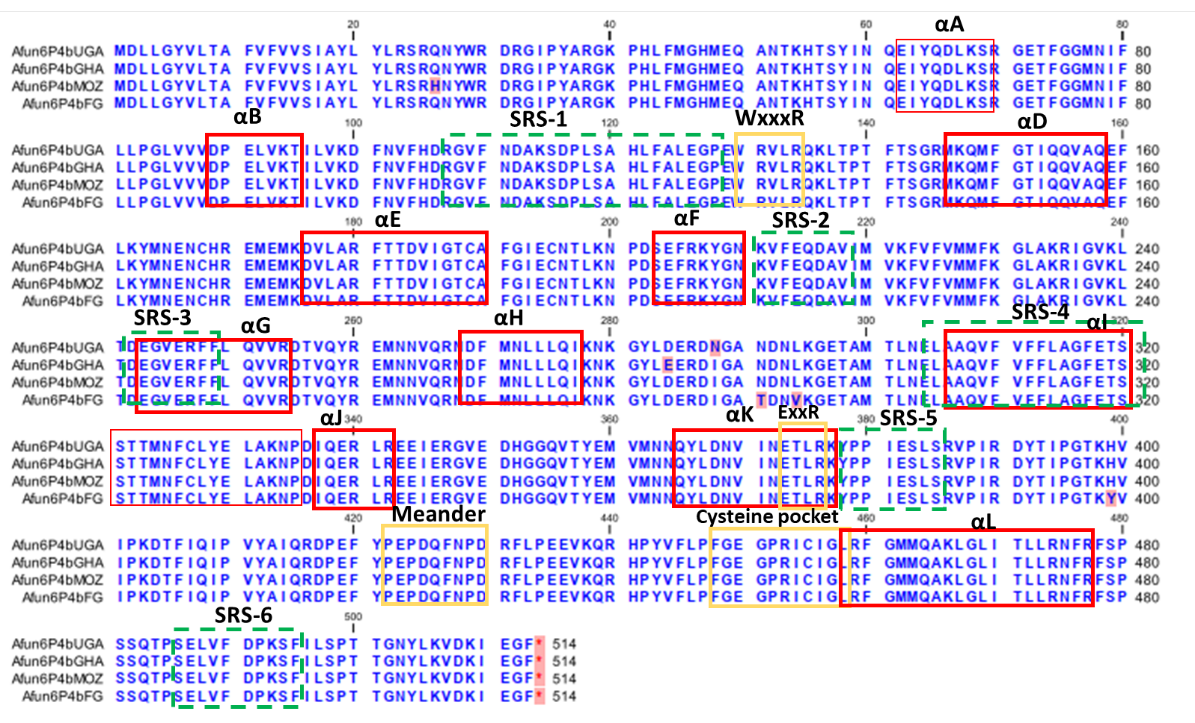
**Fig. S3B.** **A. Amino-acid alignment of *CYP6P4b* variants highlighting substitutions.** Key conserved P450 motifs and substrate recognition sites are annotated. The solid red lines represent helices A-L, while dashed blue lines correspond with the substrate recognition sites 1-6. Solid orange lines identify the structurally conserved motifs of the CYP450s. Variable residues are in hot pink. Residues 310-315 corresponds to the oxygen binding pocket. **B.** Schematic representation of haplotypes of *CYP6P4b* with D284E mutation occurring in Ghana highlighted in red.

**
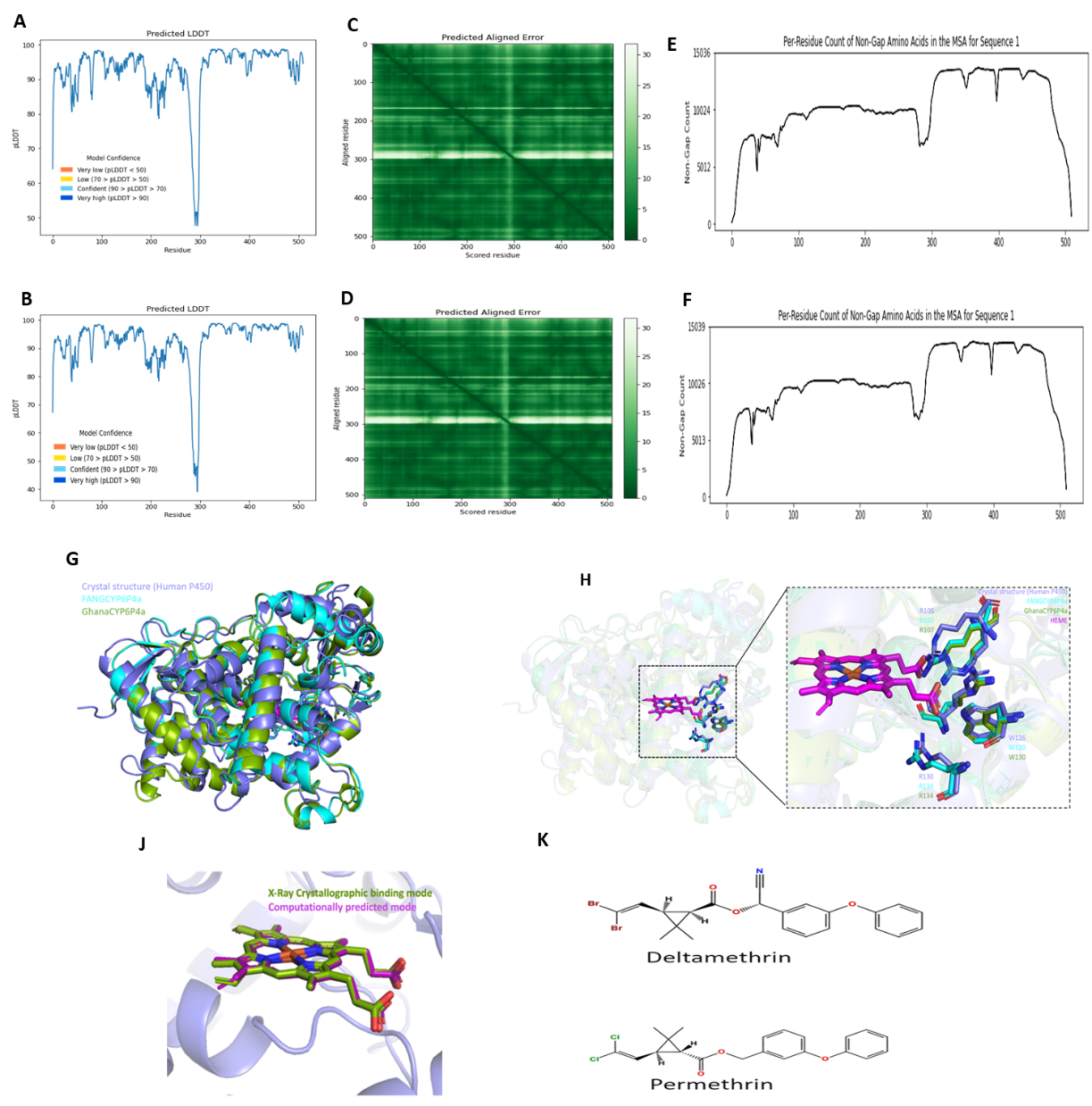
Fig. S4.** **Modelling of CYP6P4a Ghana and FANG variants.** AlphaFold’s predicted per residue accuracy (pLDDT) of modelled structures that estimates the confidence level of predictions. **A**. CYP6P4a-GHA and **B**. CYP6P4a-FANG. The Predicted aligned error reflects the relative structural prediction confidence of different domains in the structures **C**. CYP6P4a-GHA D. CYP6P4a-FANG. Multiple sequence alignments (MSA) with AlphaFold’s big fantastic database (BFD) for **E**. CYP6P4a-GHA and **F**. CYP6P4a-FANG. **G**. Structural alignment of CYP6P4a-GHA (colored green) and CYP6P4a-FANG (colored cyan) alleles, onto the crystal structure of human microsomal cytochrome P450 (colored slate blue; PDB 1TQN). **H**. Highlight of heme pocket showing optimal alignment (RMSD<0.37Å) between heme (colored purple) interacting side chain residues of the three models. **J**. Validation of docking protocol: heme molecule (colored green) from the X-ray experimental structure was re-docked into its binding pocket. The software was most accurately able to predict a heme binding mode (colored purple pink) very closely (RMSD=0.2Å) to the crystal one. **K**. 2D-structures of Deltamethrin and Permethrin


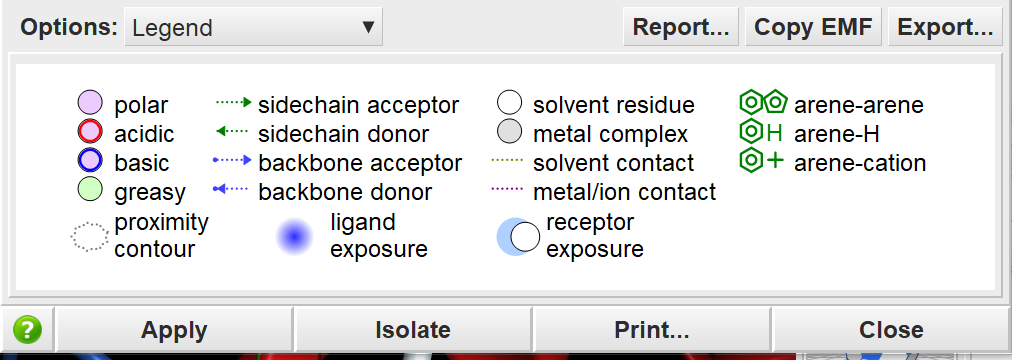

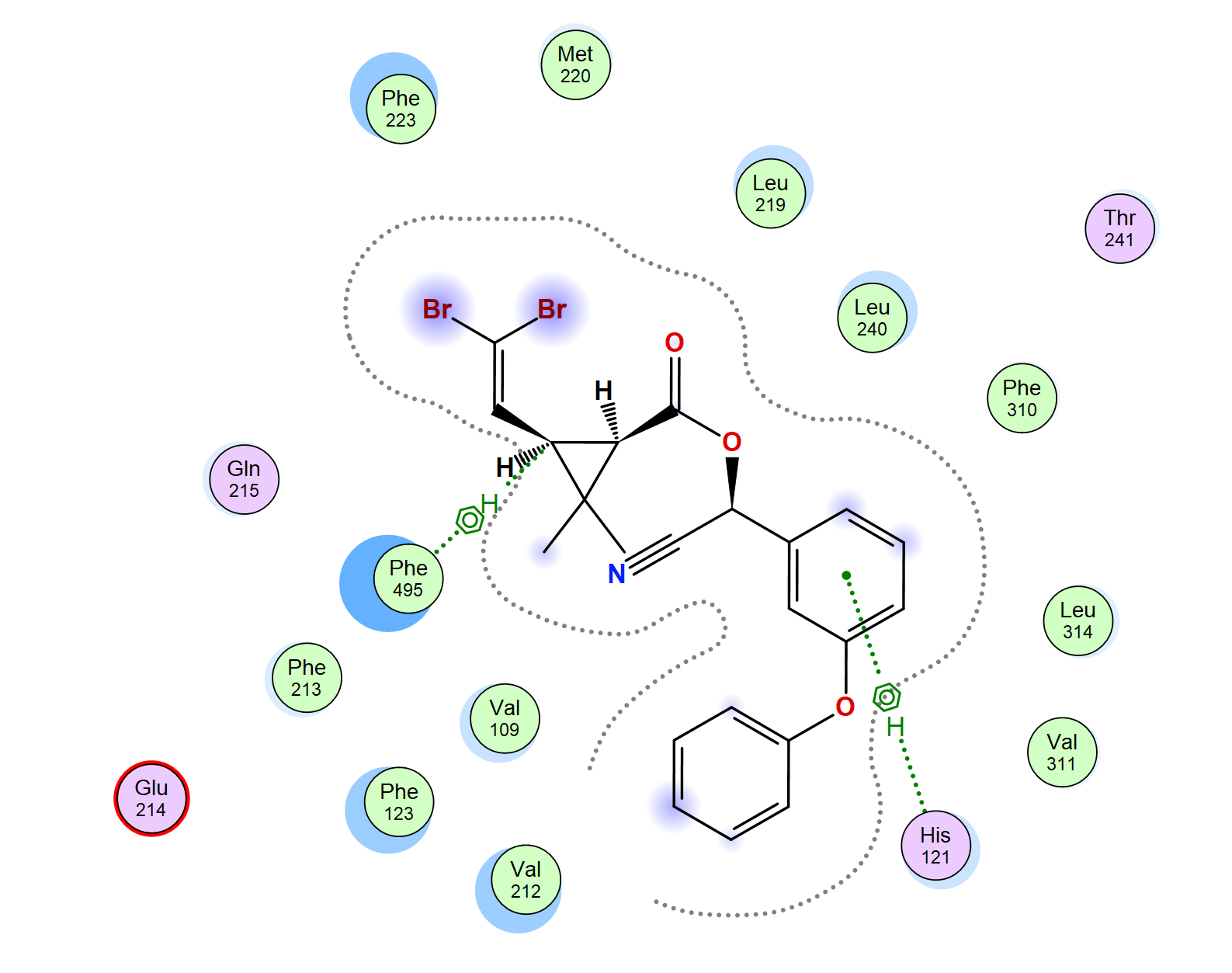

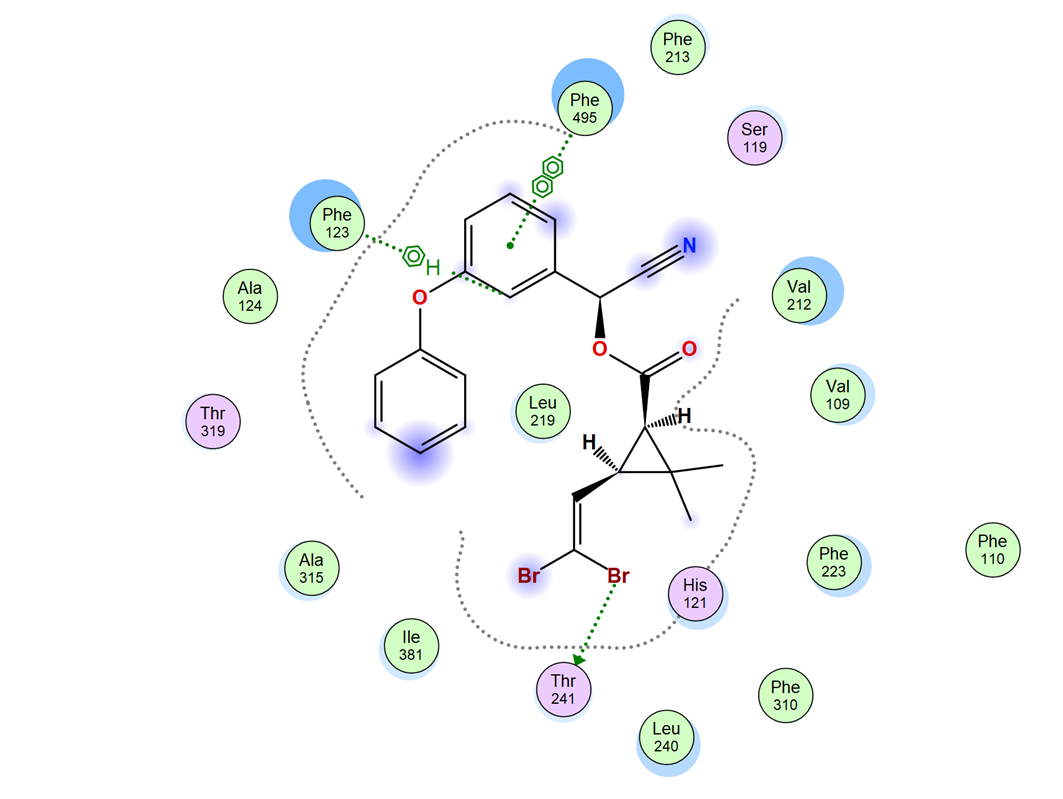

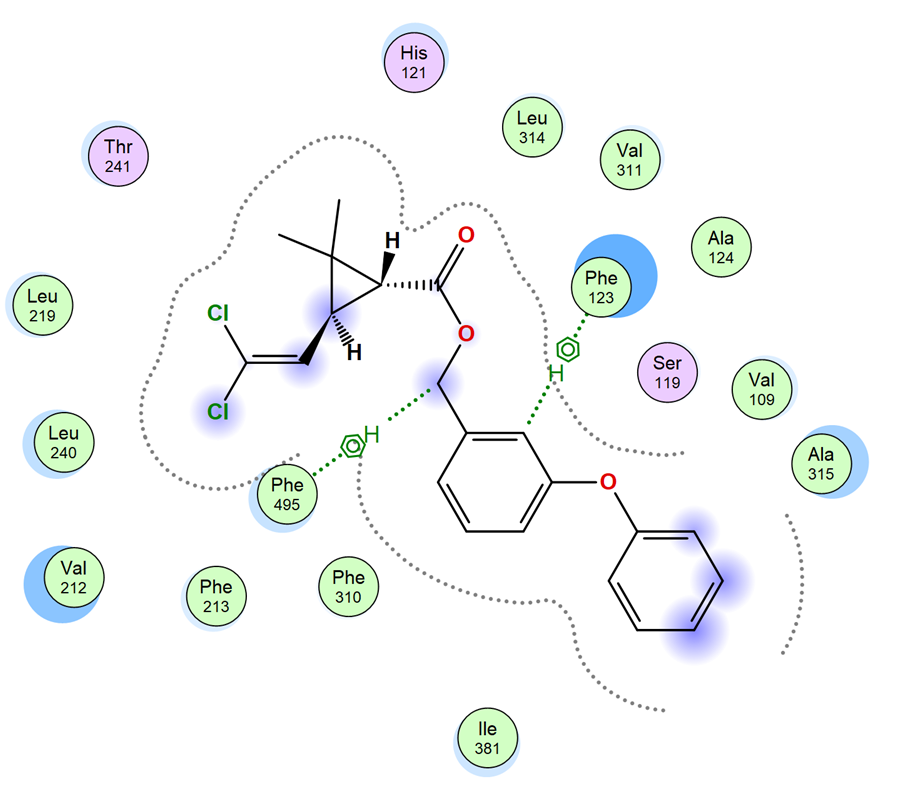

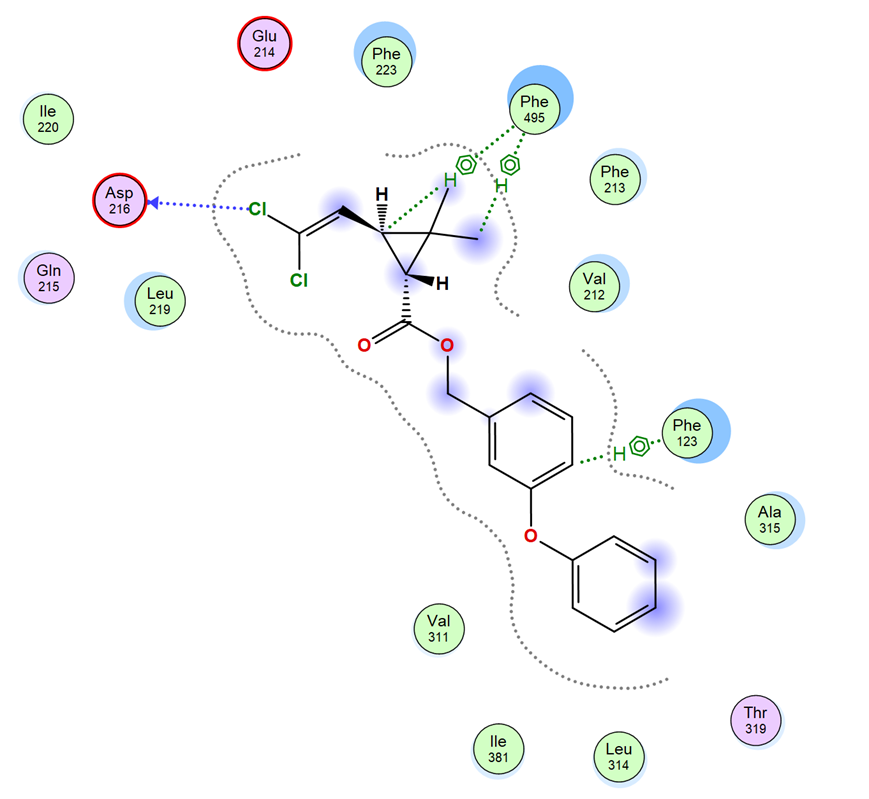


**D**

**C**

**A**

**B**

**Fig. S5. 2-D interaction map of representative pyrethroid poses with 4'-phenoxy spot approaching above the heme iron** at a distance ranging between 1.5 - 6.5Å. Deltamethrin bound to **A.** FANGCYP6P4a and **B.** GhanaCYP6P4a. 2-D interaction map of Permethrin bound to **C.** FANGCYP6P4a and **D.** GhanaCYP6P4a. Contour lines, arene-arene, arene-H, arene-cation are all estimations of van der Waals interactions. The closer the contour line to the pyrethroid, the higher the vdW clash.

**
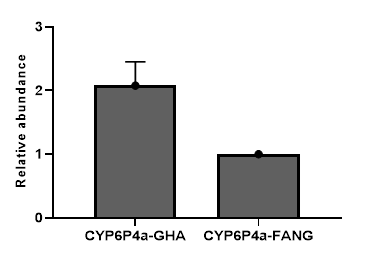
**

**Fig. S6.** **Genomic abundance of *CYP6P4a* in Ghana samples relative to abundance in FANG. Data** confirms a duplication of the gene in the Ghanian mosquitoes


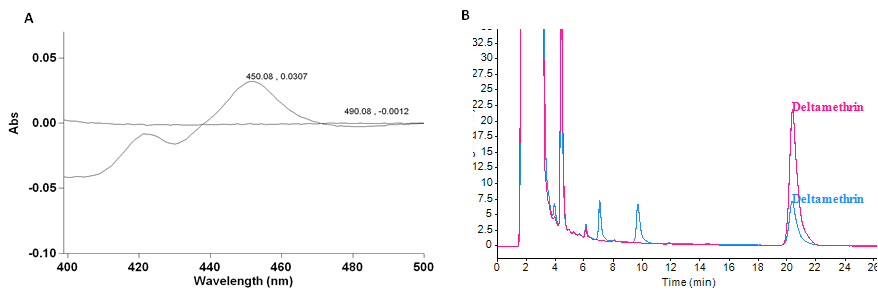


**Fig. S7.** **Heterologous expression of candidate alleles and metabolism of insecticides by recombinant CYP6P4a and CYP6P4b enzymes**. **A**. CO-difference spectrum generated from E. coli membranes expressing CYP6P4a and CYP6P4b alleles**.** Among the different allelic variants, the FANG variants *6P4a-N^286^R^289^S^291^* (6P4a-FANG) and *6P4b-T^291^V^294^Y^399^* (6P4b-FANG) demonstrated the highest expression levels, producing 15.3 µM and 7.2 µM CYP6P4a and CYP6P4b proteins, respectively. The other resistant variants produced 2-5 µM of recombinant enzymes. **B**. Overlay of HPLC chromatogram of the CYP6P4a and CYP6P4b depletion of deltamethrin with –NADPH (negative control) in hot pink and +NADPH (experimental) in blue.

**Fig. S8.** **Confirmation of transgene expression in *Drosophila melanogaster***

**D**

**C**


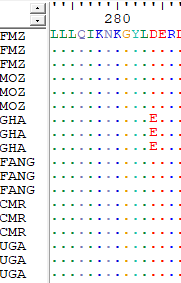

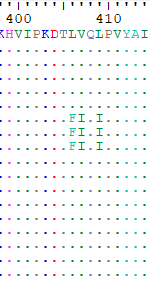

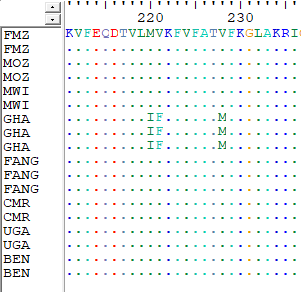


**A**

**B**

**Fig. S9.** **Schematic alignment of sequences across Africa allowing the identification of SNPs occurring in Ghana and establishment of their distribution in the field and laboratory using newly designed molecular diagnostic tools. A**. D284E mutation in Ghana and its absence in the other populations, used in the design of the CYP6P4b-D284E molecular diagnostic tool, **B.** M220I mutation in Ghana and its absence in the other populations, used in the design of the CYP6P4a-M220I molecular diagnostic tool. **C.** Frequency of resistance genotypes in Ghanaian population. **D**. Distribution of the CYP6P4b-D284E mutation in the hybrid FANG/GHANA strain showing segregation of genotypes.

**A**

**B**

**Fig. S10. Susceptibility profile of FANG/GHANA strain to evaluate impact of CYP6P4a-M220I and CYP6P4b-D284E markers** on resistance and on bio-efficacy of ITN. **E**. WHO tube assay **F**. WHO cone assay.
