# Supplementary material for: Two highly selected mutations in the tandemly duplicated CYP6P4a and CYP6P4b genes drive pyrethroid resistance in Anopheles funestus in West Africa
Source: BMC Biol. 2024 Dec 18;22:286. doi: 10.1186/s12915-024-02081-y (PMC11657943; doi:10.1186/s12915-024-02081-y)
Supplement: Supplementary file 2 — Additional file 2: Table S1. Nucleotide diversity parameters of the coding regions of CYP6P4a and CYP6P4b in An. funestus collected in 2021. Table S2 –Nucleotide diversity parameters of the coding regions of CYP6P4a and CYP6P4b in An. funestus collected in 2014. Table S3 – Characteristics of productive poses binding with pyrethroids 4'-phenoxy spot approaching above the heme iron. Table S4 – Protocol for using CYP6P4b-D284E molecular diagnostic tool. Table S5 – Protocol for using CYP6P4a-M220I molecular diagnostic tool. Table S6 – Association between insecticide susceptibility as determined by WHO tube bioassay and CYP6P4a-M220I and CYP6P4b-D284E genotypes in An. funestus crossing between field and FANG lab colony. Table S7 – Association between insecticide susceptibility as determined by WHO cone bioassay and CYP6P4a-M220I and CYP6P4b-D284E genotypes in An. funestus crossing between field and FANG lab colony. Table S8 – List of primers used in the study. [file 12915_2024_2081_MOESM2_ESM.docx]

**Supplemental Information for:**

**Two highly selected mutations in the tandemly duplicated *CYP6P4a* and *CYP6P4b* drive pyrethroid resistance in *Anopheles funestus***

Nelly M.T. Tatchou-Nebangwa^1,2*^, Leon M. J. Mugenzi^1,5^, Abdullahi Muhammad^3,7^, Derrick N. Nebangwa^4^, Mersimine F.M. Kouamo^1^, Carlos S. D. Tagne^1,6^, Theofelix A. Tekoh^1,2^, Magellan Tchouakui^1^, Stephen M. Ghogomu^2^, Sulaiman S. Ibrahim^1,8^, and Charles S. Wondji^1,3*^

^1^ Centre for Research in Infectious Diseases (CRID), P.O. BOX 13591, Yaounde, Cameroon.

^2^ Department of Biochemistry and Molecular Biology, Faculty of Science, University of Buea, P.O. Box 63, Buea, Cameroon.

^3^ Vector Biology Department, Liverpool School of Tropical Medicine (LSTM), Pembroke Place, Liverpool, L3 5QA, UK.

^4^ Randall Centre for Cell and Molecular Biophysics, Faculty of Life Sciences and Medicine, King’s College London, UK.

^5^ Syngenta Crop Protection, Werk Stein, Schaffhauserstrasse, Stein CH4332, Switzerland 6Lead.

^6^ Department of Biochemistry, Faculty of Science, University of Bamenda, Bamenda, Cameroon.

^7^ Centre for Biotechnology Research, Bayero University, Kano, PMB, 3011, Kano Nigeria.

^8^ Department of Biochemistry, Bayero University, PMB, 3011, Kano, Nigeria.

| **Sample** | | **N** | **S** | **h** | **Hd** | **Syn** | **Nsyn** |  | **π** | **D** | **D*** |
| --- | --- | --- | --- | --- | --- | --- | --- | --- | --- | --- | --- |
|  | **CYP6P4a** | | | | | | | | | | |
| **Cameroon 2021** | | 12 | 0 | 1 | 0 | 0 | 0 |  | 0 | 0 | 0 |
| **Ghana 2021** | | 9 | 38 | 4 | 0.583 | n.a | 11 |  | 0.009 | -0.013 ns | 1.104 |
| **Ghana 2014** | | 14 | 25 | 11 | 0.934 | n.a | 8 |  | 0.003 | -1.26 ns | -1.411 ns |
| **Fang** | | 6 | 20 | 6 | 1 | 15 | 5 |  | 0.007 | 1.36 ns | 1.211 |
| **Benin 2021** | | 6 | 13 | 3 | 0.733 | 10 | 3 |  | 0.004 | 0.472 ns | 0.6876 ns |
| **Uganda 2021** | | 10 | 0 | 1 | 0 | 0 | 0 |  | 0 | 0 | 0 |
| **Fumoz** | | 15 | 0 | 1 | 0 | 0 | 0 |  | 0 | 0 | 0 |
| **Moz. 2021** | | 11 | 0 | 1 | 0 | 0 | 0 |  | 0 | 0 | 0 |
| **Malawi 2021** | | 7 | 0 | 1 | 0 | 0 | 0 |  | 0 | 0 | 0 |
| **All** | | 93 | 84 | 24 | 0.782 | n.a | n.a |  | 0.012 | 0.57 | 0.89 |
|  | **CYP6P4b** | | | | | | | | | | |
| **Cameroon 2021** | | 7 | 1 | 2 | 0.286 | 1 | 0 |  | 0.00019 | -1.006 ns | -1.0488 ns |
| **Ghana 2021** | | 18 | 2 | 3 | 0.216 | 0 | 2 |  | 0.00014 | -1.508 ns | -1.989 ns |
| **Ghana 2014** | | 9 | 25 | 6 | 0.917 | 23 | 2 |  | 0.009 | 2.24 * | 1.588 * |
| **Fang** | | 11 | 24 | 6 | 0.855 | 21 | 3 |  | 0.008 | 1.965 ns | 1.398 * |
| **Uganda 2021** | | 10 | 0 | 1 | 0 | 32 | 7 |  | 0.000 | 0 | 0 |
| **Fumoz** | | 7 | 0 | 1 | 0 | 0 | 0 |  | 0 | 0 | 0 |
| **Malawi 2021** | | 9 | 0 | 1 | 0 | 0 | 0 |  | 0 | 0 | 0 |
| **All** | | 77 | 69 | 24 | 0.884 | 54 | 16 |  | 0.011 | 0.761 | -0.240 ns |

**Supplementary Tables**

**Table S1.** **Nucleotide diversity parameters of the coding regions of *CYP6P4a* and *CYP6P4b* across Africa.**

N, number of sequences; S, number of polymorphic sites; Syn, Synonymous mutations; Nsyn, Non-synonymous mutations; h, number of haplotypes; Hd, haplotype diversity; π, nucleotide diversity; D and D* Tajima’s and Fu and Li’s statistics; ns, not significant; n.a not applicable

| ***CYP6P4a* (1533bp)** | | | | | | | | | | |
| --- | --- | --- | --- | --- | --- | --- | --- | --- | --- | --- |
| **Sample** | **N** | **S** | **H** | **Hd** | **Syn** | **Nsyn** | **π** | **D** | **D*** |  |
| **FUMOZ** | 20 | 0 | 1 | 0 | 0 | 0 | 0 | 0 | 0 |  |
| **FANG** | 20 | 59 | 18 | 0.989 | 46 | 14 | 0.0179 | 2.6185** | 1.7389** |  |
| **Cameroon** | | | | | | | | | | |
| **Alive** | 20 | 48 | 20 | 1 | 38 | 10 | 0.0138 | 2.2572* | 1.5975 * |  |
| **Dead** | 20 | 47 | 20 | 1 | 37 | 10 | 0.0134 | 2.2180 * | 1.5927 ** |  |
| **All** | 40 | 50 | 35 | 1 | 39 | 11 | 0.0137 | 2.8132 ** | 1.7524 ** |  |
| **Uganda** | | | | | | | | | | |
| **Alive** | 20 | 19 | 7 | 0.8 | 16 | 3 | 0.0058 | 2.4732* | 1.2930 ns |  |
| **Dead** | 20 | 22 | 7 | 0.8 | 18 | 4 | 0.0063 | 2.0935 * | 1.3562 ns |  |
| **All** | 40 | 23 | 9 | 0.782 | 19 | 4 | 0.0059 | 2.2528 * | 1.7082** |  |
| **Malawi** | | | | | | | | | | |
| **Alive** | 20 | 0 | 1 | 0 | 0 | 0 | 0 | 0 | 0 |  |
| **Dead** | 20 | 12 | 2 | 0.189 | 8 | 4 | 0.0016 | -1.1767 ns | 1.4611 * |  |
| **All** | 40 | 12 | 2 | 0.097 | 0 | 5 | 0.0008 | -1.8035 ns | 1.4751 ns |  |
| **All countries** | | | | | | | | | | |
| **All alive** | 60 | 49 | 26 | 0.867 | 39 | 10 | 0.0127 | 2.871 ** | 1.8367 ** |  |
| **All dead** | 60 | 58 | 27 | 0.884 | 44 | 15 | 0.0132 | 2.1267 * | 2.0728 ** |  |
| **All Alive and Dead** | 120 | 61 | 43 | 0.817 | 46 | 15 | 0.0129 | 2.3161 * | 2.1068 ** |  |
| **Total all** | 160 | 78 | 61 | 0.853 | 60 | 19 | 0.0142 | 1.7492 ns | 2.3320 ** |  |
| ***CYP6P4b* (1542 bp)** | | | | | | | | | | |
| **FANG** | 20 | 48 | 16 | 0.979 | 40 | 9 | 0.0141 | 2.2307 * | 1.6020 ** |  |
| **FUMOZ** | 20 | 0 | 1 | 0 | 0 | 0 | 0 | 0 | 0 |  |
| **Cameroon** | | | | | | | | | | |
| **Alive** | 20 | 41 | 20 | 1 | 35 | 6 | 0.0118 | 2.3075 * | 1.6997 ** |  |
| **Dead** | 20 | 42 | 19 | 0.995 | 37 | 5 | 0.0115 | 1.9613 ns | 1.7027 ** |  |
| **All** | 40 | 46 | 35 | 0.994 | 41 | 6 | 0.0116 | 2.1951 * | 1.8978 ** |  |
| **Uganda** | | | | | | | | | | |
| **Alive** | 20 | 18 | 7 | 0.879 | 15 | 3 | 0.0057 | 2.7905** | 1.2681 ns |  |
| **Dead** | 20 | 25 | 10 | 0.926 | 20 | 3 | 0.0063 | 1.955 ns | 1.610 ** |  |
| **All** | 40 | 4 | 11 | 0.888 | 21 | 3 | 0.0060 | 2.0875 * | 1.4241 * |  |
| **Malawi** | | | | | | | | | | |
| **Alive** | 20 | 0 | 1 | 0 | 0 | 0 | 0 | 0 | 0 |  |
| **Dead** | 20 | 16 | 4 | 0.489 | 11 | 5 | 0.002 | -1.1706 ns | 1.2105 ns |  |
| **All** | 40 | 16 | 4 | 0.273 | 11 | 5 | 0.001 | -1.8493 ns | 1.1735 ns |  |
| **All countries** | | | | | | | | | | |
| **All alive** | 69 | 41 | 27 | 0.877 | 40 | 8 | 0.0102 | 2.4679 * | 1.9797 ** |  |
| **All dead** | 60 | 49 | 33 | 0.937 | 45 | 9 | 0.0111 | 2.0115 ns | 2.0321 ** |  |
| **All Alive and Dead** | 120 | 54 | 49 | 0.907 | 46 | 10 | 0.0106 | 1.8599 ns | 2.2632 ** |  |
| **Total all** | 160 | 64 | 65 | 0.879 | 50 | 19 | 0.0115 | 1.5198 ns | 2.4332 ** |  |

**Table S2. Nucleotide diversity parameters of Africa-wide *An. funestus* populations in 2014**

N, number of sequences; S, number of polymorphic sites; Syn, Synonymous mutations; Nsyn, Non-synonymous mutations; h, number of haplotypes; Hd, haplotype diversity; π, nucleotide diversity; D and D* Tajima’s and Fu and Li’s statistics; ns, not significant; π, n.a not applicable

**Table S3. Characteristics of productive poses binding with pyrethroids 4'-phenoxy spot approaching above the heme iron at a distance ranging between 1.5 - 6.5Å.**

| **Molecular Docking** | | |
| --- | --- | --- |
| **Deltamethrin** | **6P4a-FANG** | **6P4a-GHA** |
| 4'-phenoxy binding mode enrichment (%n) | 56% (N=25) | 71.4% (N=21) |
| Mean binding mode distance to Heme iron (Å ± SD)#* | 4.7 ± 0.38 | 3.2 ± 0.42 |
| Mean GBVI/WSA (ΔG Kcal/mol ± SD) | -34.2 ± 1.3 | -35.5 ± 0.68 |
| Mean binding mode strain energy (Kcal/mol ± SD) | 5.8 ± 1.4 | 6.05 ± 0.7 |
| Mean MM affinity (Kcal/mol ± SD)* | -8.6 ± 0.13 | -9.04 ± 0.11 |
| **Permethrin** | **6P4a-FANG** | **6P4a-GHA** |
| 4'-phenoxy binding mode enrichment (%n) | 39.1% (N=23) | 28% (N=25) |
| Mean binding mode distance to Heme iron (Å ± SD)# | 4.3 ± 0.5 | 3.4 ± 0.6 |
| Mean GBVI/WSA (ΔG Kcal/mol ± SD) | -33.5 ± 0.9 | -34.8 ± 1.2 |
| Mean binding mode strain energy (Kcal/mol ± SD) | 4.0 ± 0.67 | 4.5 ± 0.61 |
| Mean MM affinity (Kcal/mol ± SD)* | -8.17 ± 0.11 | -8.85 ± 0.28 |
| **Thermodynamics integration simulation** | | |
| **Deltamethrin** | **6P4a-FANG** | **6P4a-GHA** |
| ∆G (mmgb) | -8.1524 | -8.2958 |
| ∆Q (mol_charge) | 0 | 0 |
| ∆M (Strain) | 0 | 0 |
| **Permethrin** | **6P4a-FANG** | **6P4a-GHA** |
| ∆G (mmgb) | -7.561 | -7.6933 |
| ∆Q(mol_charge) | 0 | 0 |
| ∆M (Strain) | 0 | 0 |

N = Total number of retained docked poses out of 100 with any functional site approaching the heme iron center within distance ranging between 1.5 - 6.5Å. %n = Proportion of N poses approaching the heme iron via the 4'-phenoxy spot; the major metabolic route for pyrethroids by P450 enzymes, # = Average distance between the 4'-phenoxy spot on pyrethroid poses and the heme iron in protein binding site. GBVI/WSA = Generalized-Born Volume Integral/Weighted Surface Area energy scoring function. MM = Quantum mechanics-based forcefield * = significant p-values < 0.05 alpha level. Thermodynamics integration simulation between insecticide and modelled proteins. ∆G (mmgb) = Binding energy (∆G) based on single point molecular mechanics (mm) interaction energy. ∆Q (mol_charge) = molecules charge. ∆M(strain) = strain energy incurred.

**Table S4.**

**Protocol for using CYP6P4b-D284E molecular diagnostic tool**

**PCR Mix composition**

PCR Cycles

95°C - 5 min

94°C - 30 Sec

59°C - 30 sec

72°C - 1min 20 sec

72°C - 10 min

12°C - ∞

35 cycles

| **Component** | **Vol X1** |
| --- | --- |
| 6P4b_ARMSOF: | 0.51 µL |
| 6P4b_ARMS_CF: | 0.51 µL |
| 6P4b_ARMS_AR: | 0.51 µL |
| 6P4b_ARMS_OR: | 0.51 µL |
| Buffer A | 1.5 µL |
| dNTP mix | 0.12 µL |
| MgCl2 | 0.75 µL |
| Polymerase (Kapa taq) | 0.12 µL |
| dH2O | 9.47 µL |

-Make PCR reaction mix as indicated above,

-Dispense in PCR tubes,

-Add 1 µL DNA sample to be genotyped and run PCR using the following above

**Analysis**

-Run PCR products on 1.5% agarose gel

-Common band appears at 1284bp

-Resistant band appears at 527bp

-Susceptible band appears at 809bp (See Fig. 5A).

**Table S5.** **Protocol for using CYP6P4a-M220I molecular diagnostic tool**

| **PCR Mix composition:** |  |
| --- | --- |
| 1X Primetime Master Mix | 5.0 µL |
| 6p4a_F (10 µM) | 0.2 µL |
| 6p4a_R (10 µM) | 0.2 µL |
| LNA6p4a-Ile: Fam (10 µM) | 0.1 µL |
| LNA6p4a-Met: Hex (10 µM) | 0.1 µL |
| Molecular grade water | 3.4 µL |
| Genomic DNA | 1.0 µL |

**Amplification segment = Fast 2 step**

PCR Cycles

95°C - 10 min

95°C - 10 Sec

60°C - 45 sec

72°C - 1min 20 sec

72°C - 10 min

12°C - ∞

40 cycles (segment 2)

segment 1


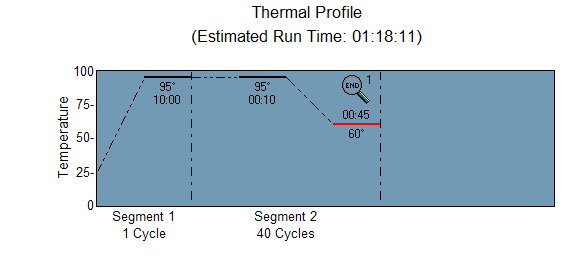


**Analysis:**

-Analysis of the genotypes is done by looking at the fluorescence for dR last.

-The FAM dye (Mutants) should be on the y-axis and HEX dye (wildtypes) on the x-axis.

-Focus on the ct values to discriminate between genotypes (See Fig. 5B).

**Table S6. Association between insecticide susceptibility as determined by WHO tube bioassay and CYP6P4a-M220I and CYP6P4b-D284E genotypes in An. funestus crossing between field and FANG lab colony.**

| **Insecticide** | **Comparison** | **OR** | **P value** | **CI** |
| --- | --- | --- | --- | --- |
| **CYP6P4b-D284E** | | | | |
| **Permethrin HS** | RR vs SS | 185 | P=0.001 | 8.1685 to 4189.8636 |
|  | RR vs RS | 6.4 | P = 0.2315 | 0.3035 to 138.4396 |
|  | RS vs SS | 39 | P = 0.0002 | 5.6823 to 267.673 |
| **Permethrin HR** | RR vs SS | 901 | P<0.0001 | 35.0574 to 23156.3417 |
|  | RR vs RS | 90 | P = 0.0001 | 9.6687 to 837.7587 |
|  | RS vs SS | 15.76 | P = 0.0675 | 0.8203 to 302.6730 |
| **Alphacypermethrin** | RR vs SS | 341 | P<0.0001 | 12.8969 to 9016.1915 |
|  | RR vs RS | 12.19 | P = 0.0228 | 1.4161 to 104.8913 |
|  | RS vs SS | 40.33 | P = 0.0126 | 2.2103 to 735.9866 |
| **CYP6P4a-M220I** | | | | |
| **Permethrin HS** | RR vs SS | 94.5 | P < 0.0001 | 12.0609 to 740.4287 |
|  | RR vs RS | 2.25 | P = 0.546 | 0.1616 to 31.3308 |
|  | RS vs SS | 42 | P = 0.0053 | 3.0337 to 581.4621 |
| **Permethrin HR** | RR vs SS | 885 | P <0.0001 | 34.4065 to 22763.8574 |
|  | RR vs RS | 44 | P = 0.0007 | 4.9920 to 387.8210 |
|  | RS vs SS | 30.39 | P = 0.0217 | 1.6472 to 560.8260 |
| **Alphacypermethrin** | RR vs SS | 189 | P = 0.0010 | 8.3163 to 4295.3047 |
|  | RR vs RS | 70.4 | P = 0.1125 | 0.7304 to 19.8691 |
|  | RS vs SS | 58.64 | P = 0.0055 | 3.8095 to 1039.3212 |
| **Genotype combination** | | | | |
| **Permethrin HR** | RR/RR vs SS/SS | 855 | P < 0.0001 | 33.2168 to 22007.6773 |
|  | RR/RS vs SS/SS | 399 | P = 0.004 | 6.7856 to 23461.6639 |
|  | RR/RR vs RS/RS | 70.4 | P = 0.0002 | 7.4829 to 662.3299 |
|  | RR/RS vs RS/RS | 21 | P = 0.055 | 0.9306 to 473.8762 |
|  | RR/RR vs RR/RS | 2.1 | P = 0.7 | 0.0720 to 63.7624 |
|  | RS/RS vs SS/SS | 19 | P = 0.05 | 0.9866 to 365.8996 |
| **Alphacypermethrin** | RR/RR vs SS/SS | 172,2 | P = 0.0012 | 7.5569 to 3923.9444 |
|  | RR/RS vs SS/SS | 90 | P = 0.0055 | 3.7550 to 2166.7357 |
|  | RR/RR vs RS/RS | 4.7 | P = 0.0704 | 0.8792 to 24.9923 |
|  | RR/RS vs RS/RS | 4.7 | P = 0.1802 | 0.4893 to 44.9061 |
|  | RR/RR vs RR/RS | 1 | P = 1.000 | 0.0721 to 13.8684 |
|  | RS/RS vs SS/SS | 43.7 | P = 0.0104 | 2.4260 to 785.2074 |

| **Bed net** | **Comparison** | **OR** | **P value** | **CI** |
| --- | --- | --- | --- | --- |
| **CYP6P4b-D284E** | | | | |
| **Permanet 2.0** | RR vs SS | 5.7273 | P = 0.0111 | 1.4901 to 22.0124 |
|  | RR vs RS | 1.0588 | P = 0.9391 | 0.2446 to 4.5830 |
|  | RS vs SS | 5.4091 | P = 0.0051 | 1.6579 to 17.6481 |
| **Olyset** | RR vs SS | 35.7778 | P<0.0001 | 6.3253 to 202.3699 |
|  | RR vs RS | 2.5667 | P = 0.2019 | 0.6034 to 10.9184 |
|  | RS vs SS | 13.95 | P = 0.0003 | 3.4023 to 57.1108 |
| **DuraNet** | RR vs SS | 19.06 | P<0.0001 | 6.4130 to 56.6413 |
|  | RR vs RS | 2.488 | P = 0.0126 | 1.241 to 4.787 |
|  | RS vs SS | 7.66 | P<0.0001 | 2.7712 to 21.1707 |
| **CYP6P4a-M220I** | | | | |
| **Permanet 2.0** | RR vs SS | 6.682 | P = 0.0205 | 1.360 to 34.35 |
|  | RR vs RS | 1.4318 | P=0.6881 | 0.2594 to 8.6736 |
|  | RS vs SS | 4.67 | P = 0.0054 | 1.6560 to 14.4989 |
| **Olyset** | RR vs SS | 27 | P<0.0001 | 4.431 to 141.3 |
|  | RR vs RS | 1.63 | P=0.5736 | 0.3591 to 8.516 |
|  | RS vs SS | 16.57 | P<0.0001 | 4.972 to 49.57 |
| **DuraNet** | RR vs SS | 10.5 | P<0.01 | 2.045 to 45.56 |
|  | RR vs RS | 2.444 | P=0.218 | 0.6379 to 9.072 |
|  | RS vs SS | 4.295 | P<0.05 | 1.289 to 13.06 |
| **Genotype combination** | | | | |
| **PermaNet 2.0** | RR/RR vs SS/SS | 6.6818 | P = 0.0317 | 1.1816 to 37.7865 |
|  | RR/RR vs RS/RS | 1.3125 | P = 0.7709 | 0.2105 to 8.1844 |
|  | RR/RR vs RR/RS | 1.167 | P = 0.892 | 0.1238 to 10.9909 |
|  | RS/RS vs SS/SS | 5.0909 | P = 0.0073 | 1.5511 to 16.7092 |
|  | RR/RS vs SS/SS | 5.7273 | P = 0.0518 | 0.9866 to 33.2482 |
|  | RR/RS vs RS/RS | 1.125 | P = 0.9 | 0.1760 to 7.1915 |
| **Olyset** | RR/RR vs SS/SS | 38.5 | P = 0.0008 | 4.5452 to 326.1145 |
|  | RR/RR vs RS/RS | 1.5556 | P = 0.6263 | 0.2627 to 9.2108 |
|  | RS/RS vs SS/SS | 24.75 | P = 0.0002 | 4.6589 to 131.4817 |
|  | RR/RR vs RR/RS | 0.438 | P = 0.534 | 0.0323 to 5.9260 |
|  | RR/RS vs SS/SS | 88 | P = 0.0005 | 6.9874 to 1108.2746 |
|  | RR/RS vs RS/RS | 3.5556 | P = 0.2669 | 0.3787 to 33.3823 |
|  | RR/RR vs RS/SS | 14 | P = 0.055 | 0.9441 to 207.6072 |
|  | RS/RS vs RS/SS | 4.5 | P = 0.12 | 0.6793 to 29.8086 |
|  | RR/RS vs RS/SS | 20 | P = 0.03 | 1.3908 to 287.6143 |
| **DuraNet** | RR/RR vs SS/SS | 10 | P = 0.0089 | 1.7809 to 56.1515 |
|  | RR/RR vs RS/RS | 2.5185 | P = 0.2223 | 0.5714 to 11.1001 |
|  | RR/RR vs RR/RS | 0.8 | P = 0.813 | 0.1257 to 5.0919 |
|  | RS/RS vs SS/SS | 3.9706 | P = 0.0357 | 1.0965 to 14.3785 |
|  | RR/RS vs SS/SS | 12.5 | P = 0.0035 | 2.2895 to 68.2473 |
|  | RR/RS vs RS/RS | 3.1481 | P=0.12 | 0.7381 to 13.4283 |

**Table S7. Association between insecticide susceptibility as determined by WHO cone bioassay and CYP6P4a-M220I and CYP6P4b-D284E genotypes in *An. funestus* crossing between field and FANG lab colony.**

**Table S8.** **List of Primers used in the study**

| **Primer ID** | **Forward primer** | **Reverse primer** |
| --- | --- | --- |
| **Full gene amplification** | | |
| CYP6P4a_full | ATGGATTTTTTGGGCTATGTGTTG | CCTTTTACACCCACCAGGAA |
|  |  |  |
| CYP6P4b_full | ATGGATCTTCTGGGTTATGTGTTG | GTATGTCCGTTCTGCACCC |
| **Cloning for in vitro heterologous expression** | | |
| ompA+2 F | GGCCGGCCATATGAAAAAGACAGCTATCGCG |  |
| ompA+2_6P4aR linker | CAACACATAGCCCAAAAAATCCATCGGAGCGGCCTGCGCTACGGTAGCGAA |  |
| ompA_6P4a_R | TCTAGAGTCGACTTAAAGCTTATCAATCTTTAG |  |
| OMPA+2_6P4b R linker | CAACACATAACCCAGAAGATCCATCGGAGCGGCCTGCGCTACGGTAGCGAA |  |
| ompA_6P4b_R | GTCGACTCTAGATCAGAAACCTTCAATCTTATCAAC |  |
| **Transgenic flies** | | |
| CYP6P4a_pUASattB | CGGCCGATGGATTTTTTGGGCTATGTGTTG | TCTAGATTAATATCAATCTTTAGATAATTTCC |
| CYP6P4b_pUASattB | CGGCCGATGGATCTTCTGGGTTATGTGTTG | TCTAGATCAGAAACCTTCAATCTTATCTACC |
| **Fly qPCR** | | |
| 6P4B_ Fly_qPCR | GGTCCACGGATTTGTATTGG | ATACGAGTTCGGACGGTGTT |
| 6P4A_ Fly_qPCR | AGCTTACGCCAACGTTTACC | GTCCCGATCACATCCGTAGT |
| RPL11 | CGATCCCTCCATCGGTATCT | AACCACTTCATGGCATCCTC |
| **CYP6P4a-M2201molecular marker** | | |
| 6p4a_F: | ATACGGCAACAAGGTGTTC | CCTTCGTCAGTCAGCTTAAC |
| LNA6p4a-Met:Hex | **TGT+TCTTA+T+G+GT+AA+A+GT** |  |
| LNA6p4a-Ile:Fam | **ACTGT+C+CTTAT+T+TT+C+AA+AT** |  |
| **CYP6P4b-D284E molecular marker** | | |
| 6P4b_ARMSOF | CTATCTGCTCATCTGTTTGCACTGGA |  |
| 6P4b_ARMSOR | TGACCATTTCATATGTCACTTGTCCAC |  |
| 6P4b_ARMS_CF | CTGCAGATTAAGAACAAAGGTTATTTGAAC |  |
| 6P4b_ARMS_AR | TTATCATTGGCTCCAATGTCACGTTTTT |  |
| **Primers used for qRT-PCR gene quantification** | | |
| CYP6P4a-qPCR | AACTCGTATTCGACCCCAAA | CGTTTCCATGGAATTACATTTTCT |
| RSP7 | GTGTTCGGTTCCAAGGTGAT | TCCGAGTTCATTTCCAGCTC |
| ACTIN | TTAAACCCAAAAGCCAATCG | ACCGGATGCATACAGTGACA |
