# Supplementary material for: Two highly selected mutations in the tandemly duplicated CYP6P4a and CYP6P4b genes drive pyrethroid resistance in Anopheles funestus in West Africa
Source: BMC Biol. 2024 Dec 18;22:286. doi: 10.1186/s12915-024-02081-y (PMC11657943; doi:10.1186/s12915-024-02081-y)
Supplement: Supplementary file 3 — Additional file 3: Method 1. Description of the amplification and cloning of full-length cDNA of An. funestus CYP6P4a and CYP6P4b. Method 2. Model structure preparation and suitability for docking. Method 3. Heterologous expression of recombinant CYP6P4a and CYP6P4b. Method 4. Description of insecticide metabolism assays and HPLC analysis. Method 5. Construction and in vivo transgenic expression of CYP6P4a and CYP6P4b in Drosophila melanogaster flies. Method 6. qPCR evaluation of transgenic expression on D. melanogaster flies. Method 7. Design of DNA diagnostic marker assays for CYP6P4a and CYP6P4b mutations. [file 12915_2024_2081_MOESM3_ESM.docx]

**Supplemental Information for:**

**Two highly selected mutations in the tandemly duplicated *CYP6P4a* and *CYP6P4b* drive pyrethroid resistance in *Anopheles funestus***

Nelly M.T. Tatchou-Nebangwa^1,2*^, Leon M. J. Mugenzi^1,5^, Abdullahi Muhammad^3,7^, Derrick N. Nebangwa^4^, Mersimine F.M. Kouamo^1^, Carlos S. D. Tagne^1,6^, Theofelix A. Tekoh^1,2^, Magellan Tchouakui^1^, Stephen M. Ghogomu^2^, Sulaiman S. Ibrahim^1,8^, and Charles S. Wondji^1,3*^

^1^ Centre for Research in Infectious Diseases (CRID), P.O. BOX 13591, Yaounde, Cameroon.

^2^ Department of Biochemistry and Molecular Biology, Faculty of Science, University of Buea, P.O. Box 63, Buea, Cameroon.

^3^ Vector Biology Department, Liverpool School of Tropical Medicine (LSTM), Pembroke Place, Liverpool, L3 5QA, UK.

^4^ Randall Centre for Cell and Molecular Biophysics, Faculty of Life Sciences and Medicine, King’s College London, UK.

^5^ Syngenta Crop Protection, Werk Stein, Schaffhauserstrasse, Stein CH4332, Switzerland 6Lead.

^6^ Department of Biochemistry, Faculty of Science, University of Bamenda, Bamenda, Cameroon.

^7^ Centre for Biotechnology Research, Bayero University, Kano, PMB, 3011, Kano Nigeria.

^8^ Department of Biochemistry, Bayero University, PMB, 3011, Kano, Nigeria.

**Methods**

1. **Amplification and cloning of full-length cDNA of *An. funestus* *CYP6P4a* and *CYP6P4b***

Total RNA from pools of 10 permethrin-resistant mosquitoes from the aforementioned origins was extracted using the PicoPure RNA isolation kit (Arcturus, Applied Biosystems, Waltham, Massachusetts, USA). The purified RNA was used for cDNA synthesis using SuperScript III (Invitrogen) with oligo-dT20 and RNase H (New England Biolabs Massachusetts, USA). The full length of the alleles of both genes were then amplified from the cDNA using Phusion Taq polymerase under the following conditions: 1 cycle at 98°C for 1 min; 35 cycles of 98°C for 10 s; 60°C for 30 s; and 72°C for 1 min and 20 s; and 1 cycle at 72°C for 10 min. The primers used are listed in Table S8. Amplicons were gel purified using the QIAquick® Gel Extraction Kit (QIAGEN, Hilden, Germany), and using the CloneJET PCR Cloning Kit, purified products were ligated to the Thermo Ficher Scientific® pJET1.2/blunt cloning vector (Thermo Ficher Scientific Waltham, Massachusetts, USA) and cloned in *E. coli* *DH5α* competent cells. Minipreparations of plasmids was done using the QIAprep® Spin Miniprep Kit (QIAGEN, Hilden, Germany) and sequenced on both strands using pJET1.2-specific primers (Microsynth AG, Switzerland).

1. **Structure preparation and suitability for docking:** Ligand and protein models were prepared for docking using structure preparation modules implemented in the Molecular Operating Environment (MOE 2021) as described previously. In brief, solvent and ligand atoms were removed from the CYP3A4 template, followed by modelling of missing residues and broken loops (S281-S286). Subsequently, all structures were protonated using default parameters, partial charges calculated for each atom, and energy minimised with gas phase parameters using the OPLS-AA [4] all atom forcefield with a gradient descent threshold of 0.001 kcal/mol. Structures of the heme ligand and corresponding binding site residues were mapped onto predicted models. Post-structural re-examinations, prepared ligand and protein files were converted to multiple formats (.pdb, .smi, .sdf) for docking.
2. **Heterologous expression of recombinant *CYP6P4a* and *CYP6P4b***

Cultured cells in LB medium were allowed to grow until reaching an optical density at 600nm of 0.7-0.8 before addition of the heme precursor δ-aminolevulinic acid (ALA), to a final concentration of 0.5 mM and isopropyl-1-thio-β-D-galactopyranoside (IPTG) to a final concentration of 1 mM were added. Membranes were isolated as done previously and P450 contents determined using spectral analysis, while CPR activities were conducted following established protocols as previously described. Briefly, about 22 h post-induction, cells were harvested and spheroplasts were prepared and sonicated. The membrane fractions containing P450s were then isolated by ultracentrifugation at 50,000g and resuspended in TSE buffer (50 mM Tris, pH 7.6, 250 mM sucrose, 10% glycerol), and stored in -80°C following measurement of P450 contents.

1. **Insecticide metabolism assays and HPLC analysis**

Insecticide metabolism assay was carried out with recombinant enzymes. The following were added to 1.5 ml tubes chilled on ice: 0.1µM of purified P450, 0.025 M potassium phosphate at pH 7.4, 0.25 mM MgCl_2_, 1 mM glucose-6-phosphate, 1 unit/mL glucose-6-phosphate dehydrogenase (G6PDH), 0.8 µM cytochrome b_5_, and 0.2 mM of test pyrethroid insecticide. The tubes were preincubated at 30°C and 1200 rpm for 5 min to activate membranes. Then, 0.1 mM NADP was added to the tubes in a final volume of 200 ml. Reactions were started and carried out at 30°C and 1200 rpm for 90 mins. All reactions were carried out in triplicate, with test reactions containing NADPH (NADPH+) and negative controls lacking NADPH (NADPH-). After 90 mins incubation, 200 μl of ice-cold acetonitrile was added to the tubes to quench reactions and the tubes incubated for 5 more min. The tubes were centrifuged at 16000g for 20 min at 4 °C, and 150 μl of supernatant were transferred to HPLC vials for the quantification of pyrethroid remaining in the samples using reverse-phase HPLC. Substrate peaks were separated with a 250 mm C18 column (Acclaim 120, Dionex) on an Agilent 1260 Infinity (Agilent, Waldbronn, Germany). Enzyme activity was quantified as percentage depletion of insecticide (difference in the amount of insecticide left) between the test (NADPH+) and control (NADPH-). Student t-test was used for the estimation of significance.

1. ***In vivo* transgenic expression of *CYP6P4a* and *CYP6P4b* in *Drosophila melanogaster* flies**

**Cloning and construction of transgenic Drosophila line**s: PCR amplification of the candidate alleles was done using minipreps that were used for *in vitro* expression above. PCR was carried out using Phusion High-Fidelity DNA Polymerase with primers having the restriction sites for *Eag*I and *Xba*I restriction enzymes (Table S8). PCR and cloning protocols were described in previous studies [6,8]. The amplicons were purified and cloned in *E. coli DH5α* competent cells using pJET1.2 vector (Thermo Ficher Scientific Waltham, Massachusetts, USA). Clones were digested with the above restriction enzymes, and the digests were ligated to pUASattB vector linearised with the above restriction enzymes. The constructs: *CYP6P4a* (UAS-CYP6P4a-GHA, and UAS-CYP6P4a-FANG) and *CYP6P4b* (UAS-CYP6P4b-GHA, UAS-CYP6P4b-FANG, and UAS-CYP6P4b-MOZ) were cloned into *E. coli DH5α* competent cells, miniprepped, and injected into the germline of *D. melanogaster* line carrying the attP40 docking site, 25C6 on chromosome 2 [y w M (eGFP, vas-int, dmRFP) ZH-2A; P{CaryP} attP40 using the PhiC31 integrase system. The process of microinjection and balancing of UAS stock to eliminate the integrase was conducted by the Fly Facility (Cambridge, UK), generating the respective transgenic lines. Ubiquitous expression of candidate alleles was obtained by crossing Actin5C-GAL4 (GAL4-Actin driver strain Act5C-GAL4, BL25374 [y[1] w[*]; P{Act5C-GAL4-w}E1/CyO, 1;2], Bloomington, IN, USA) virgin female flies with transgenic homozygote UASCYP6P4a and UAS-CYP6P4b males to produce F_1_ experimental progeny (Act5C-CYP6P4a and Act5C-CYP6P4b). For the control group, male flies with the same genetic background as the UAS transgenic lines but devoid of the pUASattB- CYP6P4a or pUASattB-CYP6P4b constructs were crossed with virgin females from the Actin5C-GAL4 driver line to generate Actin5C-GAL4-null flies.

1. **Evaluation of transgenic expression on *D. melanogaster* flies**

One microgram of total RNA from each of the three biological replicates of 5 F1 flies from the experimental and control groups was used for cDNA synthesis using SuperScript III (Invitrogen) with oligo-dT20 and RNase H, according to the manufacturer’s instructions. A serial dilution of cDNA was used to establish standard curves for each gene to assess PCR efficiency and quantitative differences between samples. The quantitative PCR (qPCR) amplification was carried out in a MX 3005 real-time PCR system (Agilent) using Brilliant III Ultra-Fast SYBR Green QPCR Master Mix (Agilent). A total of 10 ng of cDNA from each sample was used as template in a three-step program involving a denaturation at 95 °C for 3 min followed by 40 cycles of 10 s at 95 °C and 10 s at 60 °C and a last step of 1 min at 95 °C, 30 s at 55 °C, and 30 s at 95 °C. The relative expression and fold-change of each target gene in experimental groups relative to the control group was calculated according to the 2^−ΔΔCT^ method incorporating PCR efficiency after normalization with the housekeeping RPL11. All primers are provided in Table S8.

1. **Design of DNA diagnostic marker assays for *CYP6P4a* and *CYP6P4b* mutations**

Briefly, a set of four primers was designed with two outer primers: 6P4b_ARMS_OF: 5′-CTA TCT GCT CAT CTG TTT GCA CTG GA-3′ and 6P4b_ARMS_OR: 5′-GTA TGT CCG TTC TGC ACC C-3′, and two allele specific inner primers: 6P4b_ARMS_CF: 5′-CTG CAG ATT AAG AAC AAA GGT TAT TTG AAC-3′ and 6P4b_ARMS_AR: 5′- TTA TCA TTG GCT CCA ATG TCA CGT TTT T-3′ (Integrated DNA Technology, Belgium). A PCR of 35 cycles was carried out using the four primers using the conditions described in Table S4. Distinct genotype amplicons were visualised using 1.5% agarose gel electrophoresis. The homozygous wild-type allele had the S genotype band at 809 bp and the common band at 1284 bp. The homozygous mutant had the R genotype band at 527 bp and the common band at 1284 bp. The heterozygous genotype had three band comprising 527 bp, 809 bp and 1284 bp. For *CYP6P4a,* a Locked Nucleic Acid (LNA) probe-based PCR (Integrated DNA technologies, UK) was designed to discriminate between the wild-type and mutant alleles of *CYP6P4a.* The design comprises two primers: 6P4a_F 5′-ATAC GGC AAC AAG GTG TTC-3′ and 6p4a_R 5′-CCT TCG TCA GTC AGC TTA AC-3′ and two probes: the wildtype specific probe LNA6p4a-Met: Hex: TGT+TCTTA+T+G+GT+AA+A+GT and the mutant-specific probe LNA6p4a-Ile:Fam: ACTGT+C+CTTAT+T+TT+C+AA+AT (Integrated DNA Technology, Belgium). The PCR involves 10 minutes denaturation at 95°C; (Segment 1) and 40 cycles of denaturation for 10 seconds at 95°C, annealing for 45 seconds at 60°C; (Segment 2) (see Table S5 for PCR details). Distinct genotypes are identified with different fluorophores using the MxPro software <https:///www.agilent.com/en/product/real-time-pcr-(qpcr)/real-time-pcr-(qpcr)-instruments/mx3000-mx3005p-real-time-pcr-system-software>.

Detailed information on the PCR reactions can be found in Table S5.
